# Highly efficacious and safe neutralizing DNA aptamer of SARS-CoV-2 as an emerging therapy for COVID-19 disease

**DOI:** 10.1186/s12985-022-01943-7

**Published:** 2022-12-30

**Authors:** Mohamad Ammar Ayass, Trivendra Tripathi, Natalya Griko, Victor Pashkov, Jun Dai, Jin Zhang, Fabian C. Herbert, Ramya Ramankutty Nair, Tutku Okyay, Kevin Zhu, Jeremiah J. Gassensmith, Lina Abi-Mosleh

**Affiliations:** 1 Ayass Bioscience, LLC, 8501 Wade Blvd, Bldg 9, Frisco, TX 75034 USA; 2 University of Texas at Dallas, 800 W. Campbell Road, Richardson, TX 75080 USA

**Keywords:** SARS-CoV-2, COVID-19, Aptamer, VLP, Virus, Spike S protein, Safety, RBD, Toxicity, Neutralization, ACE2

## Abstract

**Background:**

The paucity of SARS-CoV-2-specific virulence factors has greatly hampered the therapeutic management of patients with COVID-19 disease. Although available vaccines and approved therapies have shown tremendous benefits, the continuous emergence of new variants of SARS-CoV-2 and side effects of existing treatments continue to challenge therapy, necessitating the development of a novel effective therapy. We have previously shown that our developed novel single-stranded DNA aptamers not only target the trimer S protein of SARS-CoV-2, but also block the interaction between ACE2 receptors and trimer S protein of Wuhan origin, Delta, Delta plus, Alpha, Lambda, Mu, and Omicron variants of SARS-CoV-2. We herein performed in vivo experiments that administer the aptamer to the lungs by intubation as well as in vitro studies utilizing PBMCs to prove the efficacy and safety of our most effective aptamer, AYA2012004_L.

**Methods:**

In vivo studies were conducted in transgenic mice expressing human ACE2 (K18hACE2), C57BL/6J, and Balb/cJ. Flow cytometry was used to check S-protein expressing pseudo-virus-like particles (VLP) uptake by the lung cells and test the immuogenicity of AYA2012004_L. Ames test was used to assess mutagenicity of AYA2012004_L. RT-PCR and histopathology were used to determine the biodistribution and toxicity of AYA2012004_L in vital organs of mice.

**Results:**

We measured the in vivo uptake of VLPs by lung cells by detecting GFP signal using flow cytometry. AYA2012004_L specifically neutralized VLP uptake and also showed no inflammatory response in mice lungs. In addition, AYA2012004_L did not induce inflammatory response in the lungs of Th1 and Th2 mouse models as well as human PBMCs. AYA2012004_L was detectable in mice lungs and noticeable in insignificant amounts in other vital organs. Accumulation of AYA2012004_L in organs decreased over time. AYA2012004_L did not induce degenerative signs in tissues as seen by histopathology and did not cause changes in the body weight of mice. Ames test also certified that AYA2012004_L is non-mutagenic and proved it to be safe for in vivo studies.

**Conclusions:**

Our aptamer is safe, effective, and can neutralize the uptake of VLPs by lung cells when administered locally suggesting that it can be used as a potential therapeutic agent for COVID-19 management.

**Supplementary Information:**

The online version contains supplementary material available at 10.1186/s12985-022-01943-7.

## Background

Severe acute respiratory syndrome coronavirus 2 (SARS-CoV-2) was reported as a new human pathogen on December 31, 2019, and its human-to-human transmission was the cause for worldwide outbreak that led to the declaration of coronavirus disease (COVID-19) pandemic. SARS-CoV-2 has continued to strain the health-care system for over two years with more than one million new infections and thousands of deaths around the world each day. COVID-19 is a major public health concern and there exists a major issue of health disparity within and between countries [1, 2]. Notably, decades of investments in life and medical sciences have accelerated a rapid scientific response with innovations in viral characterization, testing and sequencing that led to the most notable development of highly effective vaccines, which have saved many lives; however, many more remain at risk. The pandemic highlighted many challenges for developing rapid and cost-effective new therapeutic interventions with equitable delivery that prevent the development of infections and disease progression, reduce mortality and are easy to administer in all countries [3]. Therefore, the development of an appropriate and effective treatment for COVID-19 diseases is extremely urgent and critical. Research on COVID-19 treatment has focused on various virus-based and host-based therapeutics. The COVID-19 treatment guideline released by the NIH on April 1, 2022 lists Remdesivir as the only FDA-approved drug for treating COVID-19, while the FDA has emergency use authorization for Paxlovid (nirmatrelvi), molnupiravir, convalescent plasma, as well as monoclonal antibodies (more than 200 monoclonal antibodies are under development or authorized that neutralize SARS-CoV-2) for the treatment of COVID-19; however, these treatments are expensive and in short supply. In addition, convalescent plasma or monoclonal antibodies therapy requires intravenous therapy and cannot be self-administered [2, 4].

Moreover, SARS-CoV-2 is naturally mutating over time and producing new variants, some of which (Omicron, for example) proved to be more contagious and destructive than previous strains and are capable of therapeutic escape [5], ultimately creating new challenges in therapeutic approaches currently at hand.

Notably, emerging evidence during the COVID-19 pandemic era highlighted aptamers as promising theragnostic tools to treat COVID-19 infection during the rapid emergence of SARS-CoV-2 variants of concerns [6]. Thus, nucleic acid modalities including aptamers constitute a potentially effective diagnostic, prophylactic and therapeutic next-generation strategy to control COVID-19 and other diseases with their unique characteristics such as small size, high stability, low immunogenicity, advanced programmability, versatility, and safety [6–8]. Aptamers are single-stranded oligonucleotides of DNA or RNA molecules. With high specificity, sensitivity, binding affinity, and feasible alteration to the target molecule, aptamers have been deployed in many fields [9]. A group of preclinical investigations have reported that DNA aptamers [10] can inhibit the SARS-CoV-2 viral activities by targeting nucleolin, NF-Kb (nuclear factor-kappa B), GPCR-AABs, Thrombin [11] and RdRP [12]. The most favorable target for SARS-CoV-2 is the receptor binding domain (RBD) [8, 13–16] . These findings showed that aptamers have a high binding affinity to S/RBD and an inhibitory effect on S protein-ACE2 binding, implying that aptamers are an innovative strategy to treat COVID-19 disease. Recently, we have shown that our developed novel trimer S protein targeting DNA aptamer, AYA2012004_L, not only binds specifically to the trimer S protein of SARS-CoV-2, but also blocks the interaction between ACE2 receptor and trimer S protein of the Wuhan original strain as well as Delta, Delta plus, Alpha, Lambda, Mu, and Omicron variants of SARS-CoV-2. We also showed that our selected aptamer prevented S-protein expressing pseudo-SARS-CoV-2 viral like particles (VLPs) from entering ACE2 expressing host cells in vitro.

Here, we studied the neutralization of AYA2012004_L on the in vivo uptake of VLPs by mouse lung cells when administrated locally by intubation. We also studied efficacy, toxicity, mutagenicity, biodistribution, and inflammatory/immunogenicity characteristics of the same ssDNA aptamer against trimer SARS-CoV-2 S protein that was developed in our laboratory. Collectively, our data shows that AYA2012004_L is efficient, can mitigate the VLPs uptake in vivo, and is nontoxic. AYA2012004_L has a good biodistribution without accumulation in certain organs and is non-mutagenic. The data presented in this study shows that our aptamer has great efficacy in terms of preventing viral uptake in vivo and safety, suggesting that it might be a potential therapeutic agent for COVID-19 management.

## Material and methods

### Materials and reagents

Aptamer AYA2012004_L was developed by Ayass Bioscience LLC and, together with control aptamers 1 and 2, was purchased from Integrated DNA Technologies (Coralville, IA). The aptamer sequences are as follows: AYA2012004_L (TAGGGAAGAGAAGGACATATGATTTTGGGCGGGTTGAGGTGGGGGAGGAGGAGGTAGTTAGAGTTGACTAGTACATGACCACTTGA); control 1 (TAGGGAAGAGAAGGACATATGATGCGCGGTCCCGATTTGGTGTAAAATTCCCTCAGCCCTACATTGACTAGTACATGACCACTTGA); control 2 (TAGGGAAGAGAAGGACATATGATCGGTGTAGACCCTGGAGAGGGTGTGCATATGTCCGGTTGCTTGACTAGTACATGACCACTTGA). Antibodies specific to mouse CD45 PE (BioLegend), anti-human ACE2 APC (R &D System,# FAB933A), goat IgG APC (Isotype control, R &D System, # IC108A), and Fixable Viability Dye eFluor 450 (Invitrogen # 65-0863-14) were used. FluoroFix Buffer (# 422101) was purchased from BioLegend. COVID-19 S Protein/(GFP)-(6His) VLP (#VLP001) was purchased from Gentarget, Inc. RPMI 1640 (Thermo Fisher Scientific) was supplemented with 10% FCS (Gemini), 50 u/ml penicillin, 50 mg/ml streptomycin, 2 mM L-glutamate, 13 nonessential amino acids, 1 mM sodium pyruvate (SigmaAldrich), and 25 mM HEPES (Thermo Fisher Scientific). Dulbecco’s Phosphate Buffered Saline (DPBS, #1404-133) was purchased from ThermoFisher Scientific. Cell Stimulation 500$\times$ Cocktail (a cocktail of phorbol 12-myristate 13-acetate (PMA) and ionomycin, Catalog# 00-4970-93) were obtained from Invitrogen. Navios EX flow cytometer (Beckman Coulter) was used for flow cytometry experiments and flow cytometry data were analyzed with FlowJo v10 (FlowJo LLC). CpG (ODN 1826-Class B) was purchased from Invitrogen (Waltham, MA). Collagenase from clostridium histolyticum (C5138-500 MG) was purchased from Sigma-Aldrich (St. Louis, MO). Isoflurane (029405) was purchased from Covetrus (Dublin, OH). Endotracheal tubing (EZ-830EM) was purchased from E-Z Systems (Palmer, PA). Goldenrod animal lancets (GR 5 mm) were purchased from Brain Scientific, INC (Lakewood Ranch, FL).Tuberculin syringe w/detachable needle, luer-slip (#26042) was purchased from Exel International (Redondo Beach, CA). Phosphate-buffered saline (SH30256.LS) was purchased from Cytiva (Marlborough, MA).

### In vivo inhibition of VLP uptake by aptamers

Female B6.Cg-Tg(K18-ACE2)2Prlmn/J mice were randomly divided into the experimental groups for the following studies. First, to determine the in vivo dose of AYA2012004_L that is required to neutralize the uptake of 1$\times$10$^6$ VLPs, mice were given 200 *u*l of DPBS buffer containing 1 mM MgCl$_2$ alone or in combination with VLPs (1$\times$10$^6$), AYA2012004_L (10 uM), VLPs+AYA2012004_L (1 uM), VLPs+AYA2012004_L (5 uM), VLPs+AYA2012004_L (7.5 uM), and VLPs+AYA2012004_L (10 uM). Second, to determine the efficacy of modified aptamer to neutralize VLPs in vivo, mice were given 200 ul of DPBS buffer containing 1 mM MgCl$_2$ alone or in combination with VLPs (1$\times$10$^6$), VLPs+AYA2012004_L (7.5 uM), VLPs+Control Aptamer 1 (7.5 uM), and VLPs+Control Aptamer 2 (7.5 uM). Each mouse was administered with 200 ul of their respective solution via bronchoalveolar lavage, following the methodology described by others [17–19]. Briefly, mice were anesthetized and placed in a vertical position. Using the non-dominant hand, the tongue was gently placed to be removed from the mouth using the thumb finger while applying pressure on the back of the mouse with the index finger. Further, endotracheal tubing attached to a 1 ml syringe was carefully inserted into the vocal cords. Once inserted, the assembly was pushed $\approx 5$mm. The mouse was then placed in a supine position and the solution carefully administered into the lungs. In all scenarios tested, aptamers and VLPs were preincubated in solution for 1 hr prior to administration into mice. Mice were sacrificed 44 hrs post-treatment and lungs were collected for processing single cells suspension. Inhibition of VLPs uptake by aptamer was assessed by flow cytometry.

### Preparation of single cell suspension of mouse lung cells

Mice were euthanized by using a 30–70% per minute displacement of chamber air with compressed CO$_2$ and lung tissues were harvested immediately (without perfusion) and stored in 5 ml RPMI 1640 media in 15 ml conical tubes. Mice lungs were placed in an empty 60 mm cell culture dish using forceps under a sterile biosafety cabinet, and the remaining media was aspirated. The lung was shredded using sterilized surgical blades and digested by incubation with 5 ml of collagenase (1 mg/ml) dissolved in RPMI 1640 medium at 37 $^\circ$C for 30 min. Culture suspensions were stirred every 10 min, to ensure that all pieces of the lung tissue were well dislodged and did not form any clumps at the tissue trunks. Lung tissue was triturated for a single cell suspension followed by filtration through a 70 um cell strainer into a 15 ml conical tube pre-filled with 5 ml RPMI 1640 media containing 10% FBS. Cell suspension was mixed and centrifuged at 500 g at 4 $^\circ$C for 5 min. Supernatant was carefully removed and cell pellets was resuspended in 10 ml complete RPMI (cRPMI) 1640 medium containing 10% FCS (Gemini). cRPMI 1640 is composed of RPMI 1640 supplemented with 50 U/ml penicillin, 50 ug/ml streptomycin, 2 mM L-glutamate, 1X nonessential amino acids, 1 mM sodium pyruvate and 25 mM HEPES or cell staining media (2 mM EDTA DPBS containing 2% FBS).

### Immunogenic response of AYA2012004_L on mouse lung cells

Female wild-type C57BL/6J (n=3) and BALB/cJ (n=3) mice were euthanized and lung tissues were harvested and processed for single cells. Isolated lung cells cultured in cRPMI media were stimulated with or without aptamer AYA2012004_L (at various concentrations, 1.0, 3.0 and 5.0 uM) and LPS (200 ng/ml), ODN 1826 (10 uM), or LPS (100 ng/ml)+ODN 1826 (5 uM) as controls. Cells were incubated at 37 $^\circ$C for 24 and 48 hrs and at the indicated time, cell supernatant was collected by centrifugation at 200 g for 3 min and cytokine levels were assessed using the LEGENDplex Mouse Inflammation Panel (Biolegend, Catalog # 740446) according to manufacturer’s protocol. Soluble analytes were acquired using flow cytometry and analyzed with BioLegend’s LEGENDplex™.

### Immunogenic response of AYA2012004_L on human peripheral blood mononuclear cells (hPBMCs)

Human PBMCs were isolated from buffy coats (Carter BloodCare) by density-gradient centrifugation using Ficoll-Paque Plus (GE Healthcare). Human PBMCs in cRPMI media were stimulated with or without aptamer, AYA2012004_L (at various concentrations, 1, 5 and 7.5 uM) and LPS (200 ng/ml), ODN 1826 (20 uM), or LPS (100 ng/ml)+ODN 1826 (10 uM) as controls. After 24 and 48 hrs incubation, cell supernatant was collected by centrifugation at 200g for 3 min and cytokine levels were assessed using the LEGENDplex Human Inflammation Panel 1 (Biolegend, Catalog # 740808) according to manufacturer’s protocol. Soluble analytes were acquired using flow cytometry and analyzed with BioLegend’s LEGENDplex™.

### Mutagenicity of AYA2012004_L determined by the Ames test

Xenometrix Ames MPF PENTA I - with S9 and Positive Controls kit was purchased from Aniara Diagnostica(Code: AC01-512-S2-P; Manufacturer’s Part Number: C01-512-S2-P). The assay was performed in liquid media in 24-well plates during sample exposure and in 384-well plates for revertant growth and scoring. Growth, exposure, and indicator media, as well as S. typhimurium strains TA98, TA100, TA1535, TA1537 and Escherichia coli strains wp2 [pKM101] and wp2 uvrA, were included in the kit from Xenometrix. The test procedure described in the “Ames MPF Instruction for use” was followed. Briefly, bacteria were grown overnight, diluted in Exposure Medium, containing sufficient histidine (or tryptophan for E. coli) and exposed to 6 concentrations of the AYA2012004_L (0.5, 1, 2.5, 5, 7.5 and 10 uM) as well as positive and a negative control. Bacteria were incubated with AYA2012004_L and controls in 24 well-microwell plates for 90 minutes at 37 $^\circ$C with agitation to support approximately two cell divisions. The assay was performed both in the presence or absence of metabolic activation, provided by a liver homogenate S9. After 90 min, the exposure culture was diluted in pH Indicator medium lacking histidine (or tryptophan), and distributed into a 384-well plate (50 ul per well). Within two days of incubation at 37 °C, cells which have undergone the reversion to prototrophy will grow into colonies. Metabolism by the bacteria colonies lowers the pH of the medium, changing the color of the medium in that well from purple to yellow. This color change was detected visually, and the number of wells containing revertant colonies were counted at each dose and compared to a zero dose (solvent) control. We tested each dose in triplicates to allow for statistical analysis of the data.

### Distribution of AYA2012004_L in mice tissues as detected by real-time PCR assay

Female wild-type C57BL/6J mice (n=2 per group, 6-8 weeks old) were used for the study. All mice were randomly divided into the following groups: 1, 4, 8, 12, 24, and 48 hrs of treatment time. 200 ul of either DPBS buffer containing 1 mM MgCl$_2$ alone or in combination with 10 uM of AYA2012004_L was administered to each mouse. All solutions were administered via bronchoalveolar lavage. Finally, all mice were sacrificed at 1, 4, 8, 12, 24, and 48 hrs post-treatment, and their lungs, livers, kidneys, hearts, spleens, bladder, and urine were collected. Urine was collected from all subjects by gently pressing the abdominal area close to their bladder. All materials were frozen at –20 $^\circ$C immediately after their collection and then processed for nucleic acid extraction. Total DNA from a 25 mg-piece of each organ at each point of time was extracted with DNeasy Tissue Kit (Qiagen, # 69504) according to the manufacturer’s instructions. Briefly, 25 mg of the tissue were cut into small pieces and placed in a 1.5 ml centrifuge tube. 180 ul of ATL buffer and 20 ul proteinase K were added, the tube was vortexed and incubated at 56 $^\circ$C with occasional vortexing during incubation. After overnight incubation (approximately 16 hrs), 200 ul of buffer ATL was added, mixed by vortexing, and followed by the immediate addition of 200 ul of ethanol to the tube. After thorough vortexing, the mixture was pipetted into DNeasy Mini Spin column. The column was washed twice with AW1 buffer and one time with AW2 buffer, and total DNA was eluted from the column with 100 ul of nuclease free water.

### AYA2012004_L detection and quantification

Aptamer AYA2012004_L was quantitated by PCR using forward primer 5’-TAGGGAAGAGAAGGACAATGAT-3’, reverse primer 5’-TCAAGTGGTCATGTACTAGTCAA-3’ and probe 5’-[6FAM]CTCCTCCTCCCCCACCTCAA[BHQ1] ordered from Sigma-Aldrich. Quantitative PCR was performed on StepOnePlus real-time PCR System (Applied Biosystems) with TaqPath 1-Step Master Mix, CG (Thermo Fisher Scientific, cat. A15299). Twenty microliter reactions included 5 ul eluted total DNA, and the concentrations of primers and probe were 0.3 uM and 0.15 uM respectively. PCR was performed as follows: polymerase activation –95 $^\circ$C for 2 min, 40 cycles (95 $^\circ$C for 5 sec and 60 $^\circ$C for 30 sec). Estimation of aptamer molecules quantity was done by analysis of real-time PCR data by comparison with standard curve, built by using $\Delta$Ct value. $\Delta$Ct values are calculated as follows: samples for standard curve were prepared by spiking in different aptamer concentration into extracted DNA sample from control (naive) mouse. $\Delta$Ct for standard curve is the difference between Ct measured in sample with no aptamer and the Ct in a sample with known concentration of aptamer. In a similar way $\Delta$Ct value for each sample of tissue from mice administered with AYA2012004_L was obtained by subtracting this sample Ct from Ct value received from sample of tissue of control mouse, administered with the buffer alone.

### Evaluation of AYA2012004_L toxicity by histopathology

Female wild-type C57BL/6J mice were randomly divided into study groups treated with 200 $\upmu$l of DPBS buffer containing 1 mM MgCl$_2$ in the absence or presence of AYA2012004_L (5 uM) or ODN 1826 (10 ug) at day 1. Mice body weight were measured one day before treatment (on day 0) and on day 44 (24 hrs before study termination). Mice were euthanized and tissues, including lungs, brains, kidneys, hearts, lymph nodes, spleens, and livers, were harvested immediately and fixed in 10% neutral buffered formalin, embedded in paraffin blocks, and sectioned at 4 um thickness. Sections were stained with Hematoxylin and Eosin (H &E) and evaluated using light microscopy in a blinded manner by a board- certified pathologist. For histopathology, images were acquired using a Fisherbrand™ Research Grade Upright Microscope (03-000-025) equipped with a Fisherbrand™ C-Mount Digital Camera (03-000-044). Table 1 was generated from C57BL/6J WT (n=3) for each treatment group on day 45. All animals were randomly chosen.

### Cell staining

Isolated single cells from mice lungs were further stained. Briefly, $\approx$1$\times$10$^5$ lung cells were incubated with Fixable Viability Dye (1/1000 dilution with cell staining buffer) with anti-mouse CD45 or anti-human ACE2 and goat IgG at 4 °C for 30 min. Cells were washed with 2 mM EDTA DPBS two times and each time supernatant was removed by centrifugation at 200 g for 3 min two times with 2 mM EDTA DPBS. Finally, the washed cells were suspended in 500 ul of FluoroFix Buffer and subjected to flow cytometry analysis.

### Immunogenic response of lung cells to treatment with AYA2012004_L

As shown in the schematic representation of experimental design Fig. 2A, isolated single cells from mice lungs that were treated with aptamer AYA2012004_L or control aptamers 1 and 2, in the absence or presence of VLPs, were stimulated with or without 1X cocktail of phorbol 12-myristate 13-acetate (PMA) and ionomycin for 72 hrs in cRPMI media. Supernatant was collected by centrifugation at 200 g for 3 min from the cultured cells and cytokine levels were assessed using the LEGENDplex Mouse Inflammation Panel (Biolegend, Catalog # 740446) according to manufacturer’s protocol. Soluble analytes were acquired using flow cytometry and analyzed with BioLegend’s LEGENDplex™.

## Statistical analysis

Significance was determined in Prism 9.3.0 (GraphPad Software) using the unpaired Student t test for two-group comparisons and the one-way ANOVA test (Dunnett’s multiple comparisons test or Tukey’s multiple comparisons test). The *p* values <0.05 were considered statistically significant.

**Fig. 1:**
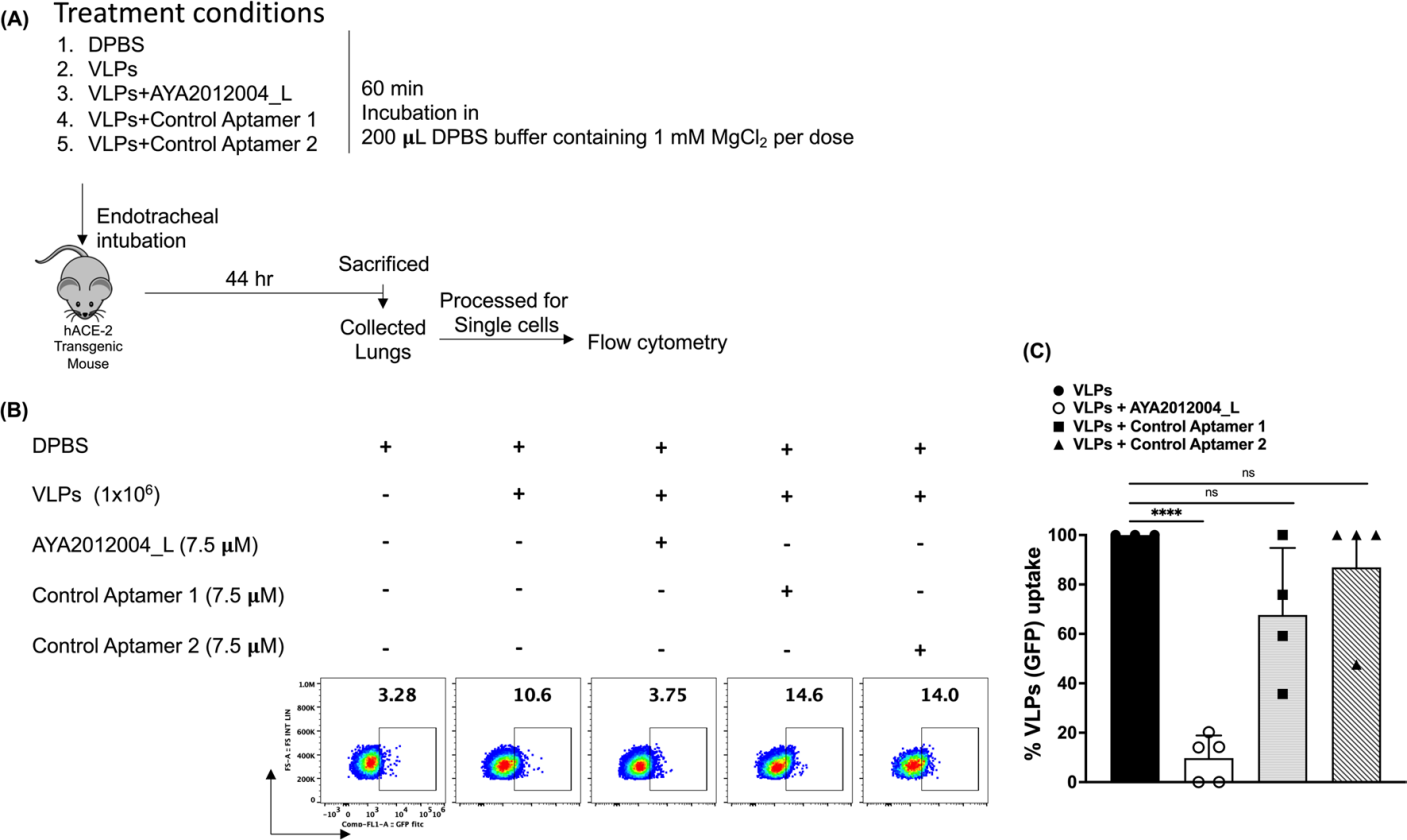
AYA2012004_L specifically neutralizes S protein VLP uptake by hACE2 transgenic mice lung cells. **A** Schematic experimental design of mice treatment conditions. **B** A representative FACS plot showing the neutralization effect of AYA2012004_L on S protein VLP uptake by lung cells. Cells were gated based on DPBS treated control group. **C** Percent uptake of S protein VLP by lung cells. Uptake of VLPs by lung cells was calculated according to the formula: [(frequency of GFP+ cells of test group − frequency of GFP+ cells of control group)/(frequency of GFP+ cells of VLPs treated group − frequency of GFP+ cells of control group)] $\times$ 100. Individual dots represent data generated with cells from different mice. Data are expressed as mean ± SD. The *p* values were determined with unpaired Student t test. **** denotes *p*$<0.0001$

## Result

### Inhibition of the uptake of S protein virus-like particles (VLPs) into lung cells of hACE2 receptor transgenic mice by AYA2012004_L

In this study, we used VLPs that have the full-length SARS-CoV-2 S protein expressed/presented on the surface of lentiviral particle to mimic the coronavirus. The VLPs are packaged with Green Fluorescent Protein (GFP) plasmid as a reporter signal. Our experiments show that when VLPs are uptaken by the lung (CD45-) cells that express hACE2 (Additional file 1: Fig. S1), GFP was expressed by the lung cells starting at 24 hrs and remained detectable up to 96 hrs post infection. Notably, we did not observe a dose-dependent increase in GFP signal due to consecutive administration of VLPs (up to three doses). Therefore, a single dose administration of the VLPs was sufficient to detect the GFP signal (Additional file 1: Fig. S2). VLPs uptake (GFP signal) was detected only by CD45- lung cells but not by the CD45+ immune cells (Additional file 1: Fig. S3). As exhibited in the schematic representation of experimental design Additional file 1: Fig. S4A, hACE2 transgenic mice were endotracheally intubated with the indicated amount of AYA2012004_L in the absence or presence of VLPs in DPBS. Mice were sacrificed at 44 hrs and lung cells were tested for their uptake of VLPs by measuring the GFP signal using flow cytometry. As exhibited in the Additional file 1: Fig. S5, gating strategy was used to determine VLP-GFP uptake by lung cells. We noticed that mice treated with mock control groups, DPBS (vehicle control) and aptamer (AYA2012004_L), demonstrate autoflorescence [20–22] by lung cells in FITC channels at an approximate frequency of 1-5%. After subtraction of the autoflorescence value at each treatment condition, our results showed that the VLPs-treated mice lung cells have a 5-fold increase in GFP signal. Of note, mice treated with VLPs in the presence of different concentration of AYA2012004_L (1, 5, 7.5, and 10 uM), showed a dose dependant decrease in GFP signal (uptake of VLPs) that was effectively diminished by 7.5 uM of AYA2012004_L. Therefore, a dose of 7.5 uM was chosen for the following experiments (Additional file 1: Fig. S4B–D). These data suggest that AYA2012004_L efficiently neutralizes the uptake of VLPs by lung cells of hACE2 receptor transgenic mice.

**Fig. 2:**
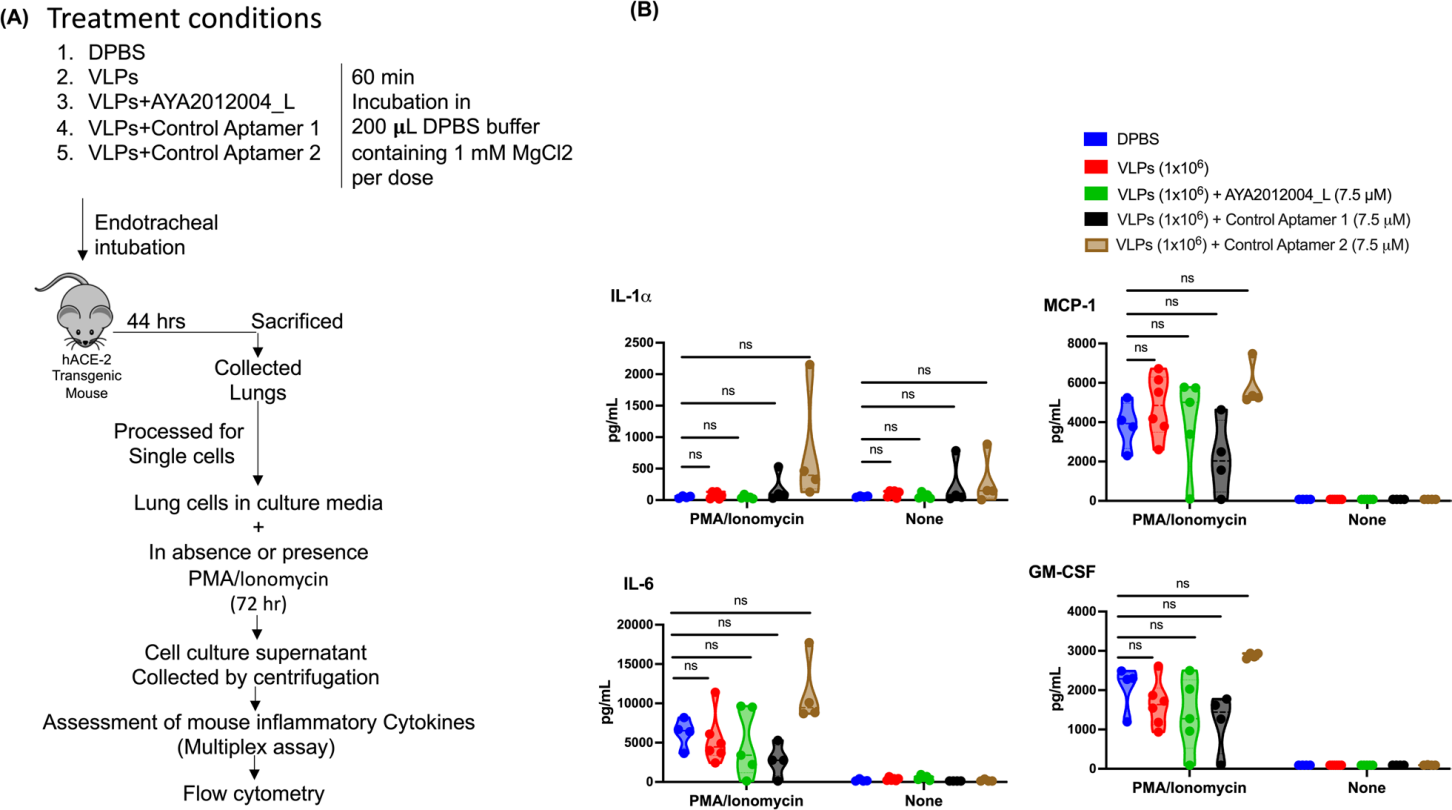
Aptamer therapy does not induce inflammatory response in hACE2 transgenic mice lungs. **A** Schematic experimental design of mice treatment conditions for evaluation of mouse inflammatory cytokines by flow cytometry. **B** Assessment of cytokines/chemokines from lung cells cultured in the absence or presence of phorbol 12-myristate 13-acetate /ionomycin was done using the LEGENDplex Mouse Inflammation Panel kit and quantitated using flow cytometry. Data were analyzed with BioLegend’s LEGENDplex™ software. Individual dots represent data generated with cells from different mice. The *p* values were determined with one-way ANOVA, Dunnett’s multiple comparisons test. ns: denotes as nonsignificant

After determining the optimal conditions for aptamer and VLP concentrations and the required incubation time for GFP signal detection in vivo, we tested the specificity and efficacy of our aptamer, AYA2012004_L, to neutralize the S-Protein expressing VLPs and prevent viral uptake in vivo. As presented in schematic experimental design Fig. 1A, hACE2 transgenic mice were endotracheally intubated with the indicated aptamer with or without VLPs in DPBS. Mice were sacrificed at 44 hrs and lung cells were tested for their uptake of VLPs by measuring GFP signal. Lung cells from VLP-treated mice displayed more than 3-fold GFP+ signal as compared to mock treated mice with DPBS. Notably, mice treated with VLPs in the presence of AYA2012004_L demonstrated a significant reduction in percentage of GFP signal (uptake of VLPs) by lung cells as compared to no aptamer or negative control aptamers treated mice (Fig. 1B, C). The data suggests that our aptamer AYA2012004_L is specific to SARS-CoV-2 S protein and is selectively neutralizing the uptake of VLPs by lung cells of hACE2 receptor transgenic mice.

### Immunogenic response of the AYA2012004_L

We next investigated the production of inflammatory cytokines by hACE2 transgenic mice lung cells that were intubated with AYA2012004_L. As shown in the schematic representation of experimental design Fig. 2A, mice were sacrificed after intubation with AYA2012004_L, and lungs were processed for single cell isolation followed by stimulation in the absence or presence of PMA/ionomycin for 72 hrs. PMA/ionomycin stimulation method is mostly used for cytokine production and in immunotoxicity assessment [23, 24]. Mouse inflammatory cytokines were measured in the supernatant of the cultured cells by the LEGENDplex multiplex assay and flow cytometry. Among the screened cytokines, GM-CSF, IL-6, MCP-1(CCL2), and IL-1$\alpha$ were detected in the lung cells of hACE2 transgenic mice, independent of the treatment condition of PMA/ionomycin. Interestingly, the levels of cytokines/chemokines tested in all the groups were not significantly different from the DPBS treated group in unstimulated (none) condition. Though in stimulated condition cytokine production was high, we could not observe significant release of cytokines/chemokines as compared to DPBS group (Fig. 2B). In contrast to IL-1$\alpha$, MCP-1(CCL2), IL-6, and GM-CSF, cytokines IL-1$\beta$, IL-10, IL-12p70, IL-17A, IL-23, IL-27, IFN-$\beta$, TNF-$\alpha$, and IFN-$\gamma$ could not be detected under any treatment condition. In conclusion, our S protein specific aptamer AYA2012004_L does not induce inflammatory response as determined by the absence of significant cytokine induction upon stimulation by PMA/ionomycin, suggesting that aptamer, AYA2012004_L, is safe for in vivo treatment since they do not induce lung inflammation.

**Fig. 3:**
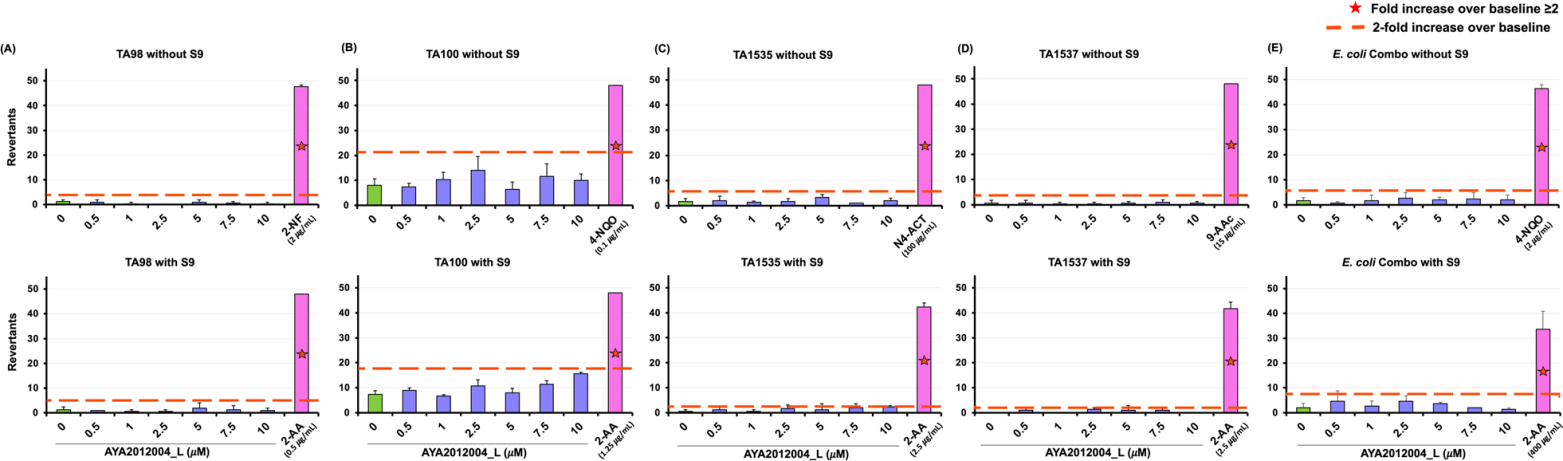
Microbial mutagenicity test for AYA2012004_L aptamer’s mutagenic potential is assessed by exposing S. typhimurium strains TA98, TA100, TA1535, TA1537 and E. coli strains wp2[pkM101] and wp2 uvrA to varying concentrations of the aptamer as well as positive and negative controls and selecting for the reversion events. AYA2012004_L was used with increasing concentrations from 0.5 to 10 uM. The assay was performed both in presence and absence of metabolic activation, provided by a liver homogenate S9. Data are a representative over three independent experiments. Data are expressed as mean ±SD

### AYA2012004_L is non-mutagenic

We determined the potential mutagenic activity of AYA2012004_L by using the Ames MPF assay. The test employs the Salmonella typhimurium strains TA98, TA100, TA1535, and TA1537, and the Escherichia coli strains wp2 [pKM101] and wp2 uvrA. Point mutations were made in the histidine (Salmonella typhimurium) or the tryptophan (E. coli) operon, rendering the bacteria incapable of producing the corresponding amino acid and therefore the bacteria cannot grow in histidine- or tryptophan- deficient media. When a mutagenic event occurs, base substitutions or frameshifts within the gene may cause a reversion to amino acid prototrophy and everted bacteria will grow in selective media that is depleted of histindine and tryptophan. TA98 and TA1537 strains are used for the detection of frameshift mutations, and T100 and TA1535 for base pair substitutions [25]. TA98 and TA100 strains are the two most commonly used strains [26, 27]. TA1535 and TA1537 are used to identify certain chemical classes of mutagens which are not detected by TA98 and TA100 [28, 29]. The E.coli strains were combined and consisted of wp2 [pKM101] and wp2 uvrA. All strains tested meet the requirements of the OECD guideline 471 for testing of chemicals. An aptamer’s mutagenic potential is assessed by exposing these amino acid -requiring organisms to varying concentrations of the aptamer and selecting for reversion events. AYA2012004_L was used with increasing concentration from 0.5 to 10 uM. We observed that all strains tested with different concentrations of AYA2012004_L show less revertants than the baseline that is defined by the positive control cut off (see Fig. 3 and Additional file 1: Table S1). Collectively these results show that aptamer AYA2012004_L is not mutagenic.

**Table 1:** Histopathology evaluation of C57BL/6 mice treated with AYA2012004_L, ODN 1826 and DPBS

Mouse tissues	AYA2012004_L	DPBS	ODN 1826
Lungs	A section of both lungs appears normal except for occasional areas of atelectasis likely introduced during dissection. Of note, no necrosis, inflammatory infiltrates, or hemorrhages are seen.	A section of both lungs shows minute areas of atelectasis as well as minute hemorrhages. These changes are likely due to tissue handling at the time of dissection. Notably, no necrosis or inflammatory infiltrates are seen.	A section of both lungs shows minute areas of atelectasis as well as minute hemorrhages. These changes are likely due to tissue handling at the time of dissection. Notably, no necrosis or inflammatory infiltrates are seen.
Brain	A sagittal section through the brain reveals normal brain architecture without areas of infarction, inflammation, or gliosis. The cortical laminations are intact, and the hippocampal cornu ammonis (CA system) and dentate gyrus are normal. The meninges and ventricular system appear unremarkable.	A sagittal section through the brain reveals multiple clear vacuoles predominately within the white matter of the brainstem and the cerebellum. These vacuoles do not appear confined to certain cells and are not involved by an associated inflammatory infiltrate. Given their random distribution within the posterior brain structures, it is reasonable to hypothesize that these vacuoles may be artifactual and due to technical manipulation during dissection. Aside from these vacuoles, the remainder of the brain is without areas of infarction, inflammation, or gliosis. The cortical laminations are intact, and the hippocampal cornu ammonis (CA system) and dentate gyrus are normal. The meninges and ventricular system appear unremarkable.	A sagittal section through the brain reveals multiple clear vacuoles predominately within the white matter of the brainstem and the cerebellum. These vacuoles do not appear confined to certain cells and are not involved by an associated inflammatory infiltrate. Given their random distribution within the posterior brain structures, it is reasonable to hypothesize that these vacuoles may be artifactual and due to technical manipulation during dissection. Aside from these vacuoles, the remainder of the brain is without areas of infarction, inflammation, or gliosis. The cortical laminations are intact, and the hippocampal cornu ammonis (CA system) and dentate gyrus are normal. The meninges and ventricular system appear unremarkable.
Kidneys	Sections of both the left and right kidneys show no histopathologic abnormalities aside from rare, mildly dilated tubules and parenchymal vessels. The glomeruli are intact and not sclerotic. The renal tubules appear healthy and are without evidence of acute tubular necrosis. A minute intraluminal cast in a collecting duct is identified, which is likely of no significance.	Sections of both the left and right kidneys show no histopathologic abnormalities aside from rare, mildly dilated tubules and parenchymal vessels. The glomeruli are intact and not sclerotic. The renal tubules appear healthy and are without evidence of acute tubular necrosis.	Sections of both the left and right kidneys show no histopathologic abnormalities aside from rare, mildly dilated tubules and parenchymal vessels. The glomeruli are intact and not sclerotic. The renal tubules appear healthy and are without evidence of acute tubular necrosis.
Heart	A section of the heart shows no histopathologic abnormalities. Notably, no areas of necrosis, infarction, inflammation, or fibrosis are identified. The valves appear normal. The ventricular and atrial septa are intact and appear normal.	A section of the heart shows no histopathologic abnormalities. Notably, no areas of necrosis, infarction, inflammation, or fibrosis are identified. The valves appear normal. The ventricular and atrial septa are intact and appear normal.	A section of the heart shows no histopathologic abnormalities. Notably, no areas of necrosis, infarction, inflammation, or fibrosis are identified. The valves appear normal. The ventricular and atrial septa are intact and appear normal.
Lymph node	A section through this tissue reveals unremarkable striated muscle. No lymphoid tissue is seen microscopically.	A section through the lymph node revealed reveals unremarkable lymphoid tissue without germinal-center formation.	A section through the lymph node revealed reveals unremarkable lymphoid tissue without germinal-center formation
Spleen	A section through the spleen reveals no histopathologic abnormalities, with good preservation of the red and white pulp. No germinal-center formation is noted	A section through the spleen reveals no histopathologic abnormalities, with good preservation of the red and white pulp. No germinal-center formation is seen.	A section through the spleen reveals no histopathologic abnormalities, with good preservation of the red and white pulp. No germinal-center formation is seen.
Liver	A section through the liver reveals normal hepatic architecture without evidence of necrosis, inflammation, or fibrosis. The portal tracts are well preserved. The hepatocytes demonstrate cytoplasmic glycogen accumulation, the amount of which is normal for ad libitum-fed mice.	A section through the liver reveals normal hepatic architecture without evidence of necrosis, inflammation, or fibrosis. The portal tracts are well preserved. The hepatocytes demonstrate cytoplasmic glycogen accumulation, the amount of which is normal for ad libitum-fed mice.	A section through the liver reveals normal hepatic architecture without evidence of necrosis, inflammation, or fibrosis. The portal tracts are well preserved. The hepatocytes demonstrate cytoplasmic glycogen accumulation, the amount of which is normal for ad libitum-fed mice.

**Fig. 4:**
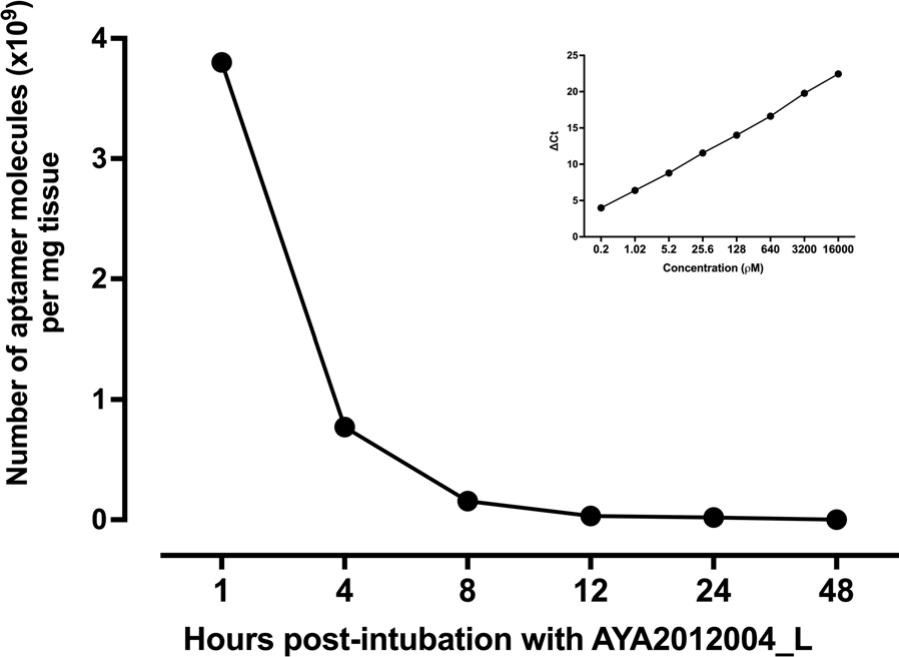
Concentration of AYA2012004_L in mouse lungs. C57BL/6J mice were treated with 200 ul of DPBS buffer containing 1 mM MgCl$_{2}$ in the absence or presence of AYA2012004_L (10 uM) via bronchoalveolar lavage. Mice were then sacrificed at 1, 4, 8, 12, 24 and 48 hrs post-treatment; and lungs were collected and processed for nucleic acid isolation. AYA2012004_L was detected in mice lungs by real-time PCR (RT-PCR) assays. Number of aptamer molecules per mg total DNA was calculated as indicated in the inset picture by plotting a standard curve between $\Delta$Ct values and total DNA concentration (pM). Data are representative over two independent experiments

### Biodistribution of AYA2012004_L

We explored the biodistribution of AYA2012004_L in mice tissues, including lungs, kidneys, livers, bladders, urine, spleens and hearts at 1, 4, 8, 12, 24, and 48 hours post-intubation. For that purpose, we extracted total DNA from 25 mg tissue of each organ or 25 ul of urine at the indicated time point and performed real-time PCR assay to detect AYA2012004_L aptamer (Fig. 4 and Additional file 1: Fig. S6). After bronchoalveolar lavage administration to lungs, high levels of AYA2012004_L was observed in lungs after one hour; the number of aptamer molecules per mg lung tissue showed a steady decrease between 1 to 8 hrs, and was completely eliminated at 48 hr. (Fig. 4). No accumulation of AYA2012004_L was observed in other organs at any time as compared to the lungs (Additional file 1: Fig. S6). The cut-off detectable $\Delta$Ct values highlighted in gray in Additional file 1: Fig. S6 denotes that $\le$0.02$\times$10$^9$ number of aptamer molecules per mg tissue was observed in kidney, liver, bladder, urine, spleen, and heart at the indicated time post-intubation. This detectable level of AYA2012004_L molecules is not considered an accountable number. Thus, the detectable but not accumulative pattern of AYA2012004_L observed in the vital organs is very close to the pattern of absorption, distribution, metabolism and excretion (ADME). Our data is in agreement with the findings of Perschbacher et al [30] who demonstrated that quantitative PCR analysis is a specific technique to detect the biodistribution and pharmacokinetics of DNA aptamers in mice. Overall, AYA2012004_L detection profiles in various tissues of mice demonstrated that AYA2012004_L in the free form is bio distributable without accumulation in any particular organ and has equal characteristics in terms of uptake into circulation, metabolism, and clearance. The highest levels of AYA2012004_L were detected in the lungs at the site of administration and showed a gradual decrease in the levels and became undetectable after 48 hrs.

**Fig. 5:**
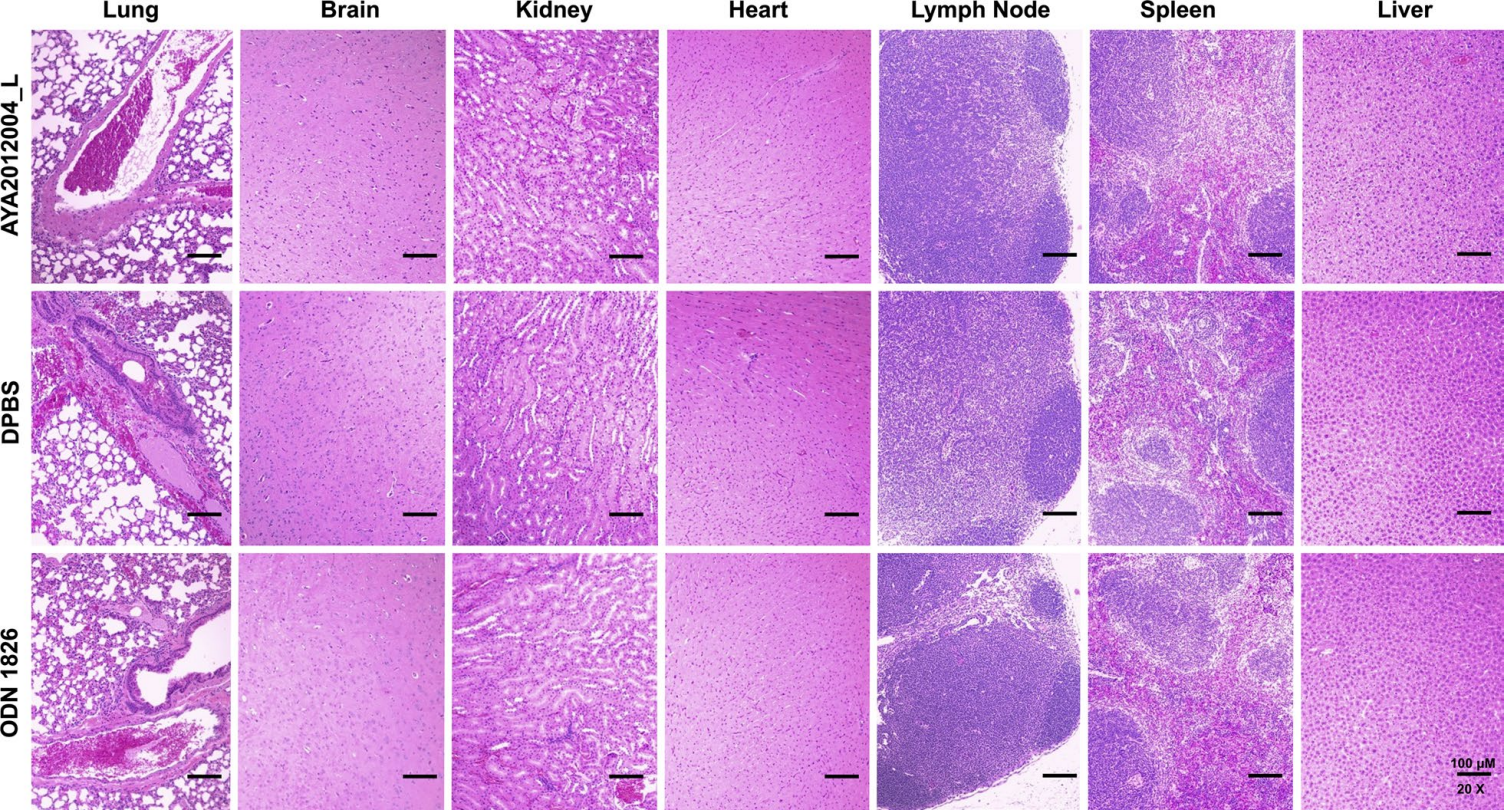
Histopathological evaluation of tissues from AYA2012004_L treated mice confirmed no toxicity to vital organs. Photomicrograph of lung, brain, kidney, heart, lymph node, spleen and liver from C57BL/6J mice administered with 200 ul of DPBS in the absence or presence of AYA2012004_L or ODN 1826 via bronchoalveolar lavage. Mice were euthanized at day 45 and tissues were harvested and processed for Hematoxylin and Eosin (H &E) staining. Histopathological evaluation of tissues were described in Table 1. Data are representative over three independent experiments

### AYA2012004_L does not induce tissue damage

Next, we addressed whether the biodistribution of aptamer AYA2012004_L induces tissue damage. ODN 1826, widely used as a vaccine adjuvant [31] and is known to protect lungs of M. tuberculosis-infected mice [32], was used as a negative control. As shown in schematic experimental design Additional file 1: Fig. S7, histopathological evaluation of C57BL/6J mice lungs, brains, kidneys, hearts, lymph nodes, spleens, and livers were assessed by H &E 45 days post AYA2012004_L administration. Under all the conditions tested and tissues investigated, there was no sign of necrosis, inflammatory infiltration or hemorrhages in all mice tissues. Most tissue sections were normal without any sign of degeneration except minute areas of atelectasis as well as minute hemorrhages due to tissue handling at the time of dissection in all treatment groups (Table 1 and Fig. 5). Similar results were observed in ODN 1826 treated mice. These results suggest that AYA2012004_L does not induce tissue degeneration or cause inflammatory infiltration. Thus, our aptamer, AYA2012004_L, is very suitable to be used in in vivo studies for therapy.

**Fig. 6:**
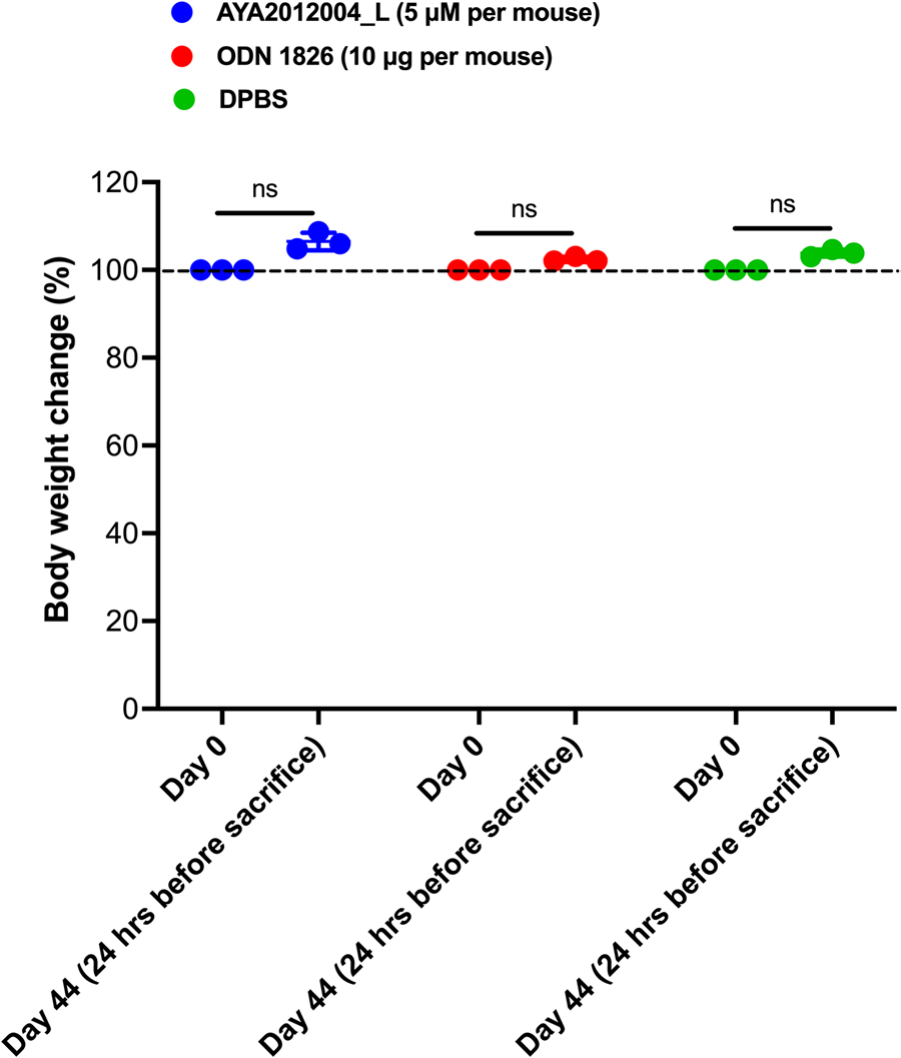
AYA2012004_L does not affect body weight of mice. C57BL/6J mice administered with 200 ul of DPBS in the absence or presence of AYA2012004_L and ODN 1826 via bronchoalveolar lavage. Mice body weight were measured one day before treatment (on day 0) and on day 44 (24 hrs before study termination). The data is expressed as a percent value of the normalization to DPBS treated mice. The *p* values were determined with one-way ANOVA, Tukey’s multiple comparisons test. ns: denotes nonsignificant.

### AYA2012004_L does not affect mouse body weight

To determine the toxicity of aptamer on mice, we monitored their body weight before and after AYA2012004_L administration. Therapies can affect many biological functions that would result in weight loss that is defined as a specific parameter in severity assessment as well as determination of humane endpoint decisions [33]. C57BL/6J mice received mock treatment of DPBS, AYA2012004_L, or ODN 1826. Body weight of mice in each group was measured on day 0 (before treatment administration) and Day 44 (24 hrs before mice were sacrificed). There was no significant difference in body weight within the treated groups on days 0 and 44 (Fig. 6) suggesting that AYA2012004_L does not elicit adverse effects leading to weight loss in mice.

**Fig. 7:**
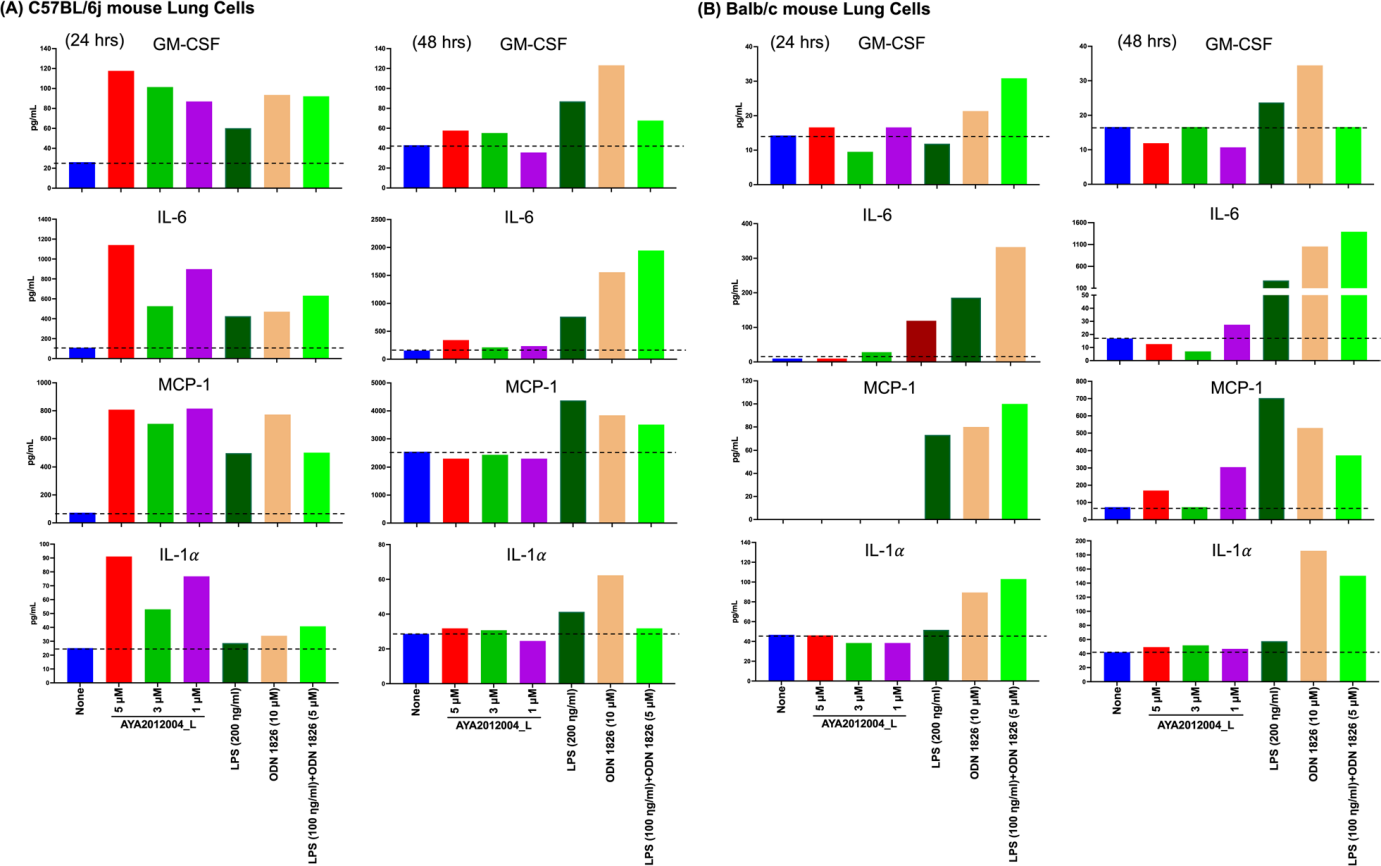
Aptamer AYA2012004_L elicits a partial immunogenic response in mice lung cells that disappeared in 48 hrs. Mice lung cells were stimulated in the presence or absence of AYA2012004_L, LPS, ODN 1826 and LPS+ODN 1826 at 37 $^\circ$C for 24 and 48 hr. Cell supernatants were collected and cytokine levels were assessed using the LEGENDplex Mouse Inflammation Panel kit. Soluble analytes were quantified using flow cytometry and analyzed with BioLegend’s LEGENDplex™ software. Data are representative over three independent experiments

### Immunogenic response to AYA2012004_L in mouse lung cells

To explore the immunogenic role of AYA2012004_L, we cultured lung cells from C57BL/6J and BALB/cJ mice (prototypical Th1 and Th2-type strains [34], respectively) with different doses of aptamer. Cytokines/chemokines release were detected at 24 and 48 hrs after incubation. Our result show that we were able to detect GM-CSF, IL-6, MCP-1(CCL2), and IL-1$\alpha$ secreted from cultured lung cells from C57BL/6J and BLAB/cj mice at the indicated incubation (Fig. 7A and B); however, cytokines, TNF-$\alpha$, IL-1$\beta$, IL-10, IL-12p70, IL-17A, IL-23, IL-27, IFN-$\beta$,and IFN-$\gamma$ secretion levels were below the assay’s limit of detection (LOD) and were therefore considered not detected (data not shown). Interestingly we noticed that cytokines secretion from C57BL/6J mice lung cells such as GM-CSF ($\approx$ 2-fold-to-5-fold increase), IL-6 ($\approx$ 2-fold-to-11-fold increase), MCP-1 ($\approx$ 2-fold-to-12-fold increase) and IL-1$\alpha$ ($\approx$ 3-fold-to-4-fold increase) were increased upon AYA2012004_L at 24 hrs stimulation at all concentrations as compared to mock (none, no aptamer treatment) but the levels of these cytokines went back to normal(comparable to mock) after 48 hrs (Fig. 7A). Notably, BALB/cJ mice lung cells showed comparable cytokines (GM-CSF, IL-6, MCP-1(CCL2) and IL-1$\alpha$) release on different doses of aptamers when compared to mock at 24 and 48 hrs after incubation (Fig. 7B). We noticed that cytokines (GM-CSF, IL-6, MCP-1(CCL2) and IL-1) induced by AYA2012004_L in C57BL/6J or BALB/cJ mice lung cells were attenuated as compared to ODN 1826 and LPS or ODN 1826 + LPS especially after 48 hrs. At the 24 hr time point, there was an increase in the level of the same cytokines in C57BL/6J mice lung cells that are treated with AYA2012004_L as compared to Balb/cJ mice. However the levels of these cytokines returned to baseline at the 48 hr time point and remained comparable to Balb/cJ. Liu et al. [35] demonstrated that naive C57BL/6J mice contained more-mature subsets of dendritic cells than naive Balb/cJ mice. Thus, differences in the reactivities of dendritic cells to aptamer treatment through TLRs may determine the susceptibility and resistance of C57BL/6J (Th1 model) and Balb/cJ (Th2 model) mice for immune stimulation. It is possible that the endocytosis of nucleic acid aptamers by dendritic cells, monocytes, macrophages, B lymphocytes, and other immune cells can trigger several key cytokines; however, the levels of these cytokines would subside over a short period of time and do not cause any permanent damage. This is consistent with our data showing that AYA2012004_L partially induced immune stimulation in the Th1 mouse model that disappeared after 48 hrs, suggesting that AYA2012004_L is partially an immune stimulant without tissue damage.

**Fig. 8:**
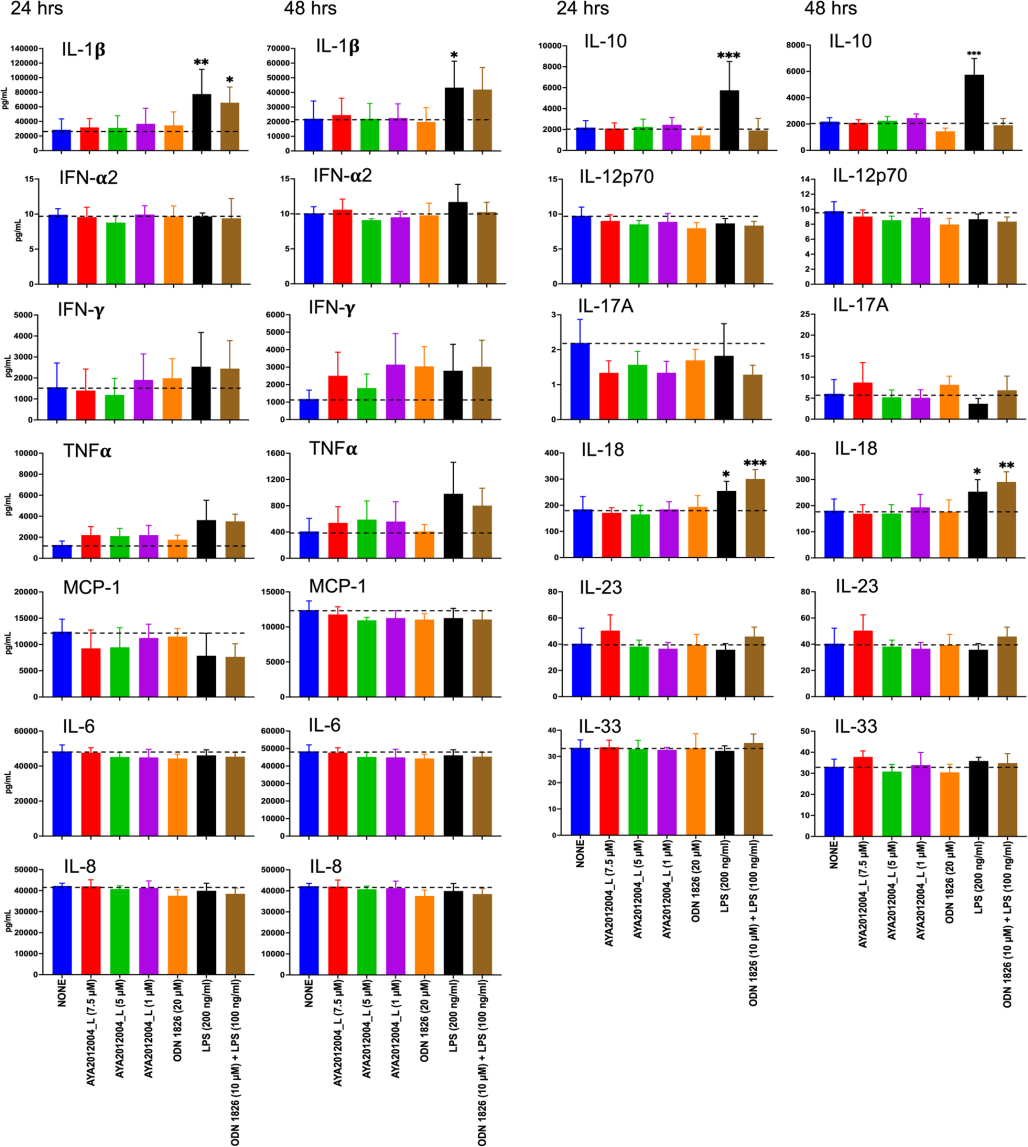
Aptamer AYA2012004_L does not elicit an immunogenic response in human peripheral blood mononuclear cells (hPBMCs). Human PBMCs were stimulated with or without AYA2012004_L, LPS, ODN 1826 and LPS+ODN 1826 at 37 $^\circ$C for 24 and 48 h. The amount of cytokines secreted from PBMCs were assessed by the LEGENDplex Human Inflammation kit. Soluble analytes were quantified using flow cytometry and analyzed with BioLegend’s LEGENDplex™ software. The *p* values were determined with one-way ANOVA, Dunnett’s multiple comparison test. * denotes $p<$0.05, ** denotes $p<$0.01 and *** denotes *p*<0.001

### Immunogenic response to AYA2012004_L by human PBMCs

To address the possible immunogenicity of AYA2012004_L aptamer in human model, we measured the amount of human inflammatory cytokines/chemokines, IL-1$\beta$, IFN-$\alpha$2, IFN-$\gamma$, TNF-$\alpha$, MCP-1 (CCL2), IL-6, IL-8 (CXCL8), IL-10, IL-12p70, IL-17A, IL-18, IL-23, and IL-33 that are induced by different doses of AYA2012004_L in human PBMCs. Control groups included cells that were either mock, ODN 1826, LPS and LPS+ODN 1826 treated. Human PBMCs were incubated with the indicated condition and media was collected 24 and 48 hrs post treatment as shown in Fig. 8. PBMC cells treated with 1, 5, or 7.5 uM AYA2012004_L showed comparable secretion of IL-1$\beta$, IFN-$\alpha$2, MCP-1 (CCL2), IL-6, IL-8 (CXCL8), IL-10, IL-12p70, IL-17A, IL-18, IL-23, and IL-33 to mock or ODN 1826 treated cells at 24 and 48 hrs. Under the same conditions, TNF-$\alpha$ levels showed a non-significant increase at 24 and 48 hrs and IFN-$\gamma$ after 48 hrs of stimulation with AYA2012004_L as compared to the mock and ODN 1826 treated human PBMC at all concentrations tested. Positive controls, mainly LPS showed increased production of IL-1$\beta$, IL-10, and IL-18 at 24 and 48 hrs of the stimulation at the indicated dose as compared to mock or AYA2012004_L treated cells. Collectively, these results suggest that aptamer, AYA2012004_L, is not an immune stimulator, and are in agreement with the findings of Thiel et al. [36] who demonstrated that smooth muscle cell-targeted RNA aptamer did not increase inflammatory cytokines, IL-6, IFN-$\beta$, and IFN-$\gamma$ release from human PBMCs. Together, our studies suggest that AYA2012004_L has an acceptable safety profile.

## Discussion

Like other viruses, SARS-CoV-2 has already shown a high rate of genetic mutation leading to the emergence of new variants. Some mutations modified the target site of antibodies or drugs, leaving them ineffective in the treatment of COVID-19 disease [37, 38]. Although vaccines and approved anti-SARS-CoV-2 therapy have proven to be beneficial, the continued emergence of new variants still challenges public health and medical therapy. The need for improving anti-SARS-CoV-2 therapies with fewer side effects have led to the fast development of targeted therapies such as aptamers. With the goal of developing a neutralizing aptamer(s) for SARS-CoV-2, we have shown that a series of ssDNA aptamers obtained by SELEX method can recognize the universal S protein of SARS-CoV-2 variants including wild type, Delta, Delta+, Mu, Lambda, Alpha, and Omicron. Additionally, our aptamers in vitro precisely neutralize the uptake of SARS-CoV-2 S protein expressing virus-like particles (VLPs) by HEK 293T cells that over express ACE2 receptor. In this study, we evaluated the efficacy and safety of the reported aptamer, AYA2012004_L, in transgenic mice expressing human ACE2 by the human cytokeratin 18 promoter (K18 hACE2) as well as Balb/cJ and C57BL/6J mice models, in vivo and in vitro. The results showed that AYA2012004_L binds to S protein expressed on VLPs and effectively neutralizes/diminishes the uptake of VLPs by lung cells of human ACE2 transgenic mice. One limitation is the lack of fluorescence microscopy images for lung sections showing the GFP signal. Although fluorescence microscopy and flow cytometry can be used to identify cell types and quantify fluorescence, we elected to analyze the GFP signal using flow cytometry in order to capture and quantitate the signal in a large number of CD45- cells. Meanwhile, we assessed the toxicity of AYA2012004_L in mice models by analyzing their effect on tissues histology as well as body weight changes. Immunogenic response by mice lung cells or by human PBMCs during aptamer treatment was also addressed. Our results demonstrated that AYA2012004_L has significant anti-SARS-CoV-2 neutralizing efficacy without systemic toxicity that is commonly seen with other anti-SARS-CoV-2 drugs [39–41]. The histopathological investigation also showed that no pathological changes were observed in vital organs including lungs, kidneys, spleens, hearts, livers, brains, and lymph nodes. These results strongly suggest that our aptamer can suppress the attachment of SARS-CoV-2 to host cells and obstruct the entry of SARS-CoV-2 virus into lung cells, thereby impeding viral replication without induction of innate immunity. Other studies [8, 13–16] have reported aptamer binding to S protein and their ability to inhibit the interaction between S protein and ACE2 receptors. However, to the best of our knowledge, this is the first work that shows DNA aptamer’s binding and inhibitory activities to S protein of SARS-CoV-2 in k18-hACE2 mice in vivo study. K18-hACE2 mice express ACE2 regulated by the human cytokeratin 18(k18) promoter in airway epithelial cells. This animal model has been widely used for assessing therapeutic interventions and pathogenesis of mild and lethal COVID-19 [42, 43]. Also, this is the first study that utilizes SARS-CoV-2 pseudo-VLPs with the k18-hACE2 mice and it shows that the mouse model is suitable for in vivo testing of SARS-CoV-2 targeted neutralizing agents, small molecules and antibodies. Therefore, the use of k18-hACE2 provides strong evidence of the stated efficacy against SARS-CoV-2. This is also the first study that administers therapy by intubation locally to the primary site of infection without the need for intravenous injection suggesting that in humans, AYA2012004_L can be administered as an inhaler or nebulizer without the need for invasive methods like antibody therapy by infusion.

Aptamers have many features including relatively small physical size, flexible structure, quick chemical production, versatile chemical modification, high stability, and lack of immunogenicity that make them promising agents for treatment of COVID-19 [6, 7, 44–47]. Our aptamer did not induce mutagenesis in bacterial strains using the Ames test, providing further proof of the safety of utilizing aptamers for therapy. Over the past two years SARS-CoV-2 has emerged several variants with spike mutations that facilitate immune escape, raising many questions on the efficacy of available vaccines and anti-SARS-CoV-2 monoclonal antibody therapies [48–51]. Meanwhile notable characteristics of aptamers, such as ease of modification as the virus mutates, makes aptamers promising therapeutic targets for COVID-19 [6, 7]. Notably, aptamers can be synthesized chemically in a relatively short time for a fraction of the cost as compared to other anti-COVID-19 drugs. Aptamers have high cell and tissue permeability due to their small size [44, 52]. In our study, we observed that AYA2012004_L in the free form, is biodistributable without accumulation in the vital organs of mice and was detectable in mice tissue sites belonging to circulation, metabolism, and clearance. We also used comprehensive immunogenicity and histological methods to provide evidence of the AYA2012004_L induced a partial immunogenic response that disappeared after 48 hrs and non/minimal toxicity in the mouse models. Additionally, AYA2012004_L, when administered locally using intubation, showed that it can remain in the lung cells up to 24 hrs after administration, which exhibits some therapeutic characteristics of slow distribution from the central compartment and slow renal filtration and elimination [53].

## Conclusion

In conclusion, we have demonstrated that the reported aptamer AYA2012004_L can be administered locally using intubation and is efficiently internalized and can inhibit the interaction between the S protein expressed on VLPs and ACE2 receptors on mice lung cells, without induction of cellular toxicity, host immunogenic responses or alteration of gene expression profiling. Therefore, the findings suggest that the reported aptamer, AYA2012004_L, is an effective treatment for SARS-CoV-2 infection. Our aptamer can be pharmaceutically useful for in vivo therapeutic purposes and can be administered locally using aerosolization, such as nebulization or inhaler, without the need for intravenous administration of antibodies.

## Supplementary Information

**Additional file 1: Fig. S1**. Expression of hACE2 receptors on lung cells (CD45- Cells) in hACE2 Transgenic mice. Lung cells were isolated from female B6.Cg-Tg(K18-ACE2)2Prlmn/J mice, female C57BL/6J and female BALB/cJ. Cells were stained with Fixable Viability Dye, anti-mouse CD45, anti-human ACE-2 and/or goat IgG. Cells were gated based on isotype staining (Goat IgG). Expression of hACE2 receptor on mice lung cells was assessed by flow cytometry analysis. **Fig. S2**. S protein mediated VLP uptake by lung cells of hACE2 Transgenic mice. 1x10^6^ VLP was administered to female B6.Cg-Tg(K18-ACE2)2Prlmn/J mice in 200 of DPBS buffer containing 1 mM MgCl2 via bronchoalveolar lavage on 2 hrs intervals as 1, 3, and 6 injections. Mice were sacrificed after 24 and 96 hrs, and lung cells were analyzed by flow cytometry to detect GFP signal from VLP in FITC channel. Individual dots represent data generated with cells from different mice. **Fig. S3**. Expression of S protein VLP uptake by live lung cells (CD45- cells) in hACE2 Transgenic mice. Female B6.Cg-Tg(K18-ACE2)2Prlmn/J mice were administered with 1x10^6^ S protein VLPs in 200 of DPBS buffer containing 1 mM MgCl_2_ via bronchoalveolar lavage on 2 hrs interval as 1, 3 and 6 injections. Mice were sacrificed after 24 and 96 hrs; and cells were stained with Fixable Viability Dye, anti-mouse CD45, anti-human ACE2 and/or goat IgG. Cells were gated based on isotype staining (Goat IgG). Expression of hACE2 receptor and S protein VLP uptake by mice lung cells were assessed by flow cytometry analysis. **Fig. S4**. Aptamer induced dose-dependent neutralization of S protein VLP uptake by lung cells of hACE2 transgenic mice. **A** Schematic experimental design of mice treatment with or without AYA2012004_L. **B** A representative FACS plot showing the neutralization effect of AYA2012004_L on the S protein VLP uptake by lung cells. Cells were gated based on DPBS treated group as control. **C** Summarized data for the frequency of GFP+ cells (uptake of S protein VLP) and **D** Summarized data for the percent uptake of S protein VLP by lung cells under every treatment condition. Uptake of S protein VLP by lung cells was calculated according to the formula: [(frequency of GFP+ cells of test group − frequency of GFP+ cells of control group)/(frequency of GFP+ cells of S protein VLP treated group − frequency of GFP+ cells of control group)] × 100. Individual dots represent data generated with cells from different mice (n=3). Data are expressed as mean ± SD. The* p* values were determined with one way ANOVA test (Dunnett’s multiple comparison test). *p < 0.05, **p < 0.01, ***p < 0.001 and ****p < 0.0001. **Fig. S5**. Gating strategy for GFP-VLP signal. Lungs were processed for single-cell isolation. Single cells from the lungs were gated for GFP signal (uptake of VLP-GFP) by the CD45-Live cells. Lung cells were stained with anti-mouse CD45 PE and fixable viability dye. Stained cells were acquired for three colors flow cytometry. Cells were gated first on FS-A and SS-A and then gated for single cells that were further gated for CD45- Live cells (Lung cells). Live lung cells (CD45- Live cells) were then gated from GFP. This gating strategy is applied in Figure 1 and Figure S5.** Fig. S6**. Detection of AYA2012004_L in mouse vital organs and urine by RT-PCR. C57BL/6J mice were administered with 200 l of DPBS buffer containing 1 mM MgCl2 in the absence or presence of AYA2012004_L (10 M) via bronchoalveolar lavage. Mice were then sacrificed at 1, 4, 8, 12, 24 and 48 hrs post-treatment. Lungs, livers, kidneys, hearts, spleens, bladder and urine were collected and processed for nucleic acid isolation. AYA2012004_L was detected in mice tissues by real time polymerase chain reaction (RT-PCR) assays. Grey color area is the cut off ∆Ct value in the organs of AYA2012004_L that demonstrates 0.02x10^9^ number of aptamer molecules per mg total DNA as calculated. The data are mean±SD of two independent experiments.** Fig. S7**. A schematic experimental design for histopathology analysis of mice tissues administered with 200 ul of DPBS buffer containing 1 mM MgCl2 in the absence or presence of AYA2012004_L (5 mM) or ODN 1826 (20 g) at day 1 via bronchoalveolar lavage. Mice body weight were measured one day before treatment (on day 0) and on day 44 (24 hrs before study termination). On day 45, mice were euthanized, and tissues were harvested and processed for Haemotoxylin and Eosin (H&E).** Table S1**. Assessment of the mutagenic potential of AYA2012004_L.

## Data Availability

All data generated or analyzed during this study are included in this published article [and its additional files].

## Abbreviations

COVID-19: Coronavirus disease 2019
SARS-CoV-2: Severe acute respiratory syndrome coronavirus 2
RBD: Receptor binding domain
ACE2: Angiotensin-converting enzyme 2
SELEX: Systematic evolution of ligands by exponential enrichment
VLPs: Virus-like particles
GFP: Green fluorescent protein
PBMCs: Peripheral blood mononuclear cells
