# Supplementary material for: Highly efficacious and safe neutralizing DNA aptamer of SARS-CoV-2 as an emerging therapy for COVID-19 disease
Source: Virol J. 2022 Dec 30;19:227. doi: 10.1186/s12985-022-01943-7 (PMC9800238; doi:10.1186/s12985-022-01943-7)
Supplement: Supplementary file 1 — Additional file 1: Fig. S1. Expression of hACE2 receptors on lung cells (CD45- Cells) in hACE2 Transgenic mice. Lung cells were isolated from female B6.Cg-Tg(K18-ACE2)2Prlmn/J mice, female C57BL/6J and female BALB/cJ. Cells were stained with Fixable Viability Dye, anti-mouse CD45, anti-human ACE-2 and/or goat IgG. Cells were gated based on isotype staining (Goat IgG). Expression of hACE2 receptor on mice lung cells was assessed by flow cytometry analysis. Fig. S2. S protein mediated VLP uptake by lung cells of hACE2 Transgenic mice. 1x10^6^ VLP was administered to female B6.Cg-Tg(K18-ACE2)2Prlmn/J mice in 200 of DPBS buffer containing 1 mM MgCl2 via bronchoalveolar lavage on 2 hrs intervals as 1, 3, and 6 injections. Mice were sacrificed after 24 and 96 hrs, and lung cells were analyzed by flow cytometry to detect GFP signal from VLP in FITC channel. Individual dots represent data generated with cells from different mice. Fig. S3. Expression of S protein VLP uptake by live lung cells (CD45- cells) in hACE2 Transgenic mice. Female B6.Cg-Tg(K18-ACE2)2Prlmn/J mice were administered with 1x10^6^ S protein VLPs in 200 of DPBS buffer containing 1 mM MgCl2 via bronchoalveolar lavage on 2 hrs interval as 1, 3 and 6 injections. Mice were sacrificed after 24 and 96 hrs; and cells were stained with Fixable Viability Dye, anti-mouse CD45, anti-human ACE2 and/or goat IgG. Cells were gated based on isotype staining (Goat IgG). Expression of hACE2 receptor and S protein VLP uptake by mice lung cells were assessed by flow cytometry analysis. Fig. S4. Aptamer induced dose-dependent neutralization of S protein VLP uptake by lung cells of hACE2 transgenic mice. A Schematic experimental design of mice treatment with or without AYA2012004_L. B A representative FACS plot showing the neutralization effect of AYA2012004_L on the S protein VLP uptake by lung cells. Cells were gated based on DPBS treated group as control. C Summarized data for the frequency of GFP+ cells (uptake of S protein VLP [file 12985_2022_1943_MOESM1_ESM.docx]

Supplementary figures and tables:


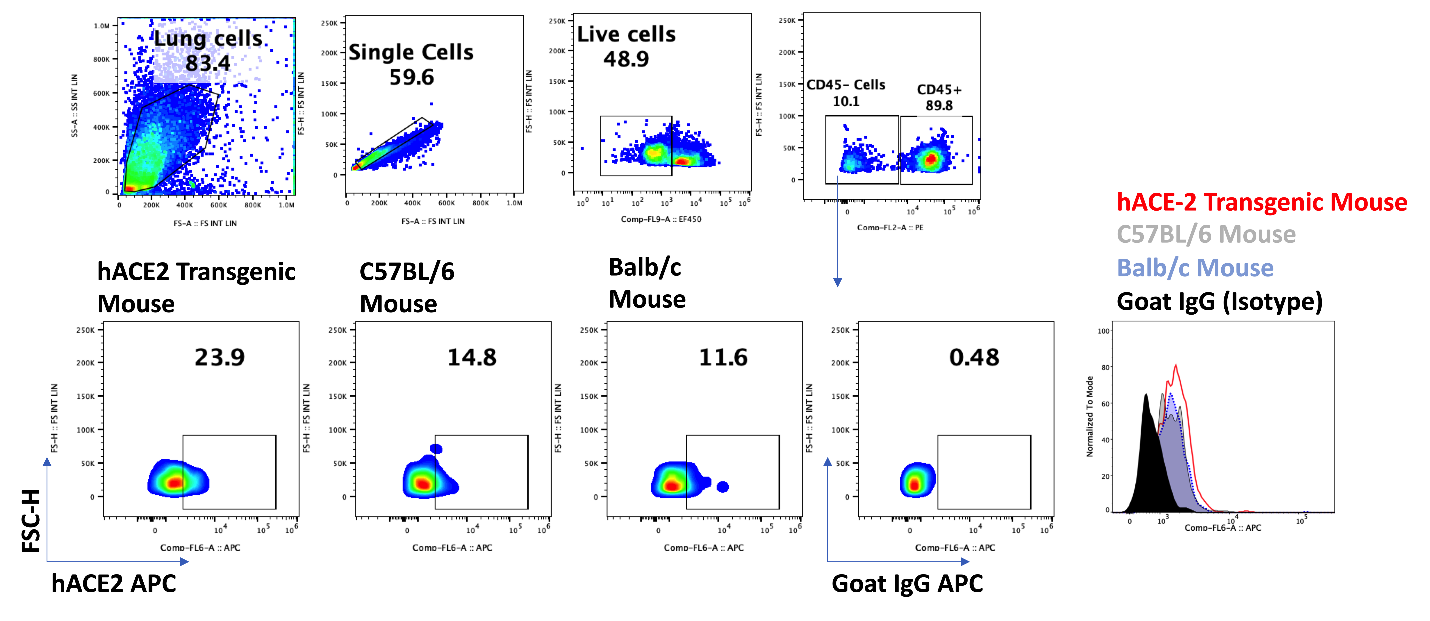


**Figure S1.** Expression of hACE2 receptors on lung cells (CD45- Cells) in hACE2 Transgenic mice. Lung cells were isolated from female B6.Cg-Tg(K18-ACE2)2Prlmn/J mice, female C57BL/6J and female BALB/cJ. Cells were stained with Fixable Viability Dye, anti-mouse CD45, anti-human ACE-2 and/or goat IgG. Cells were gated based on isotype staining (Goat IgG). Expression of hACE2 receptor on mice lung cells was assessed by flow cytometry analysis.


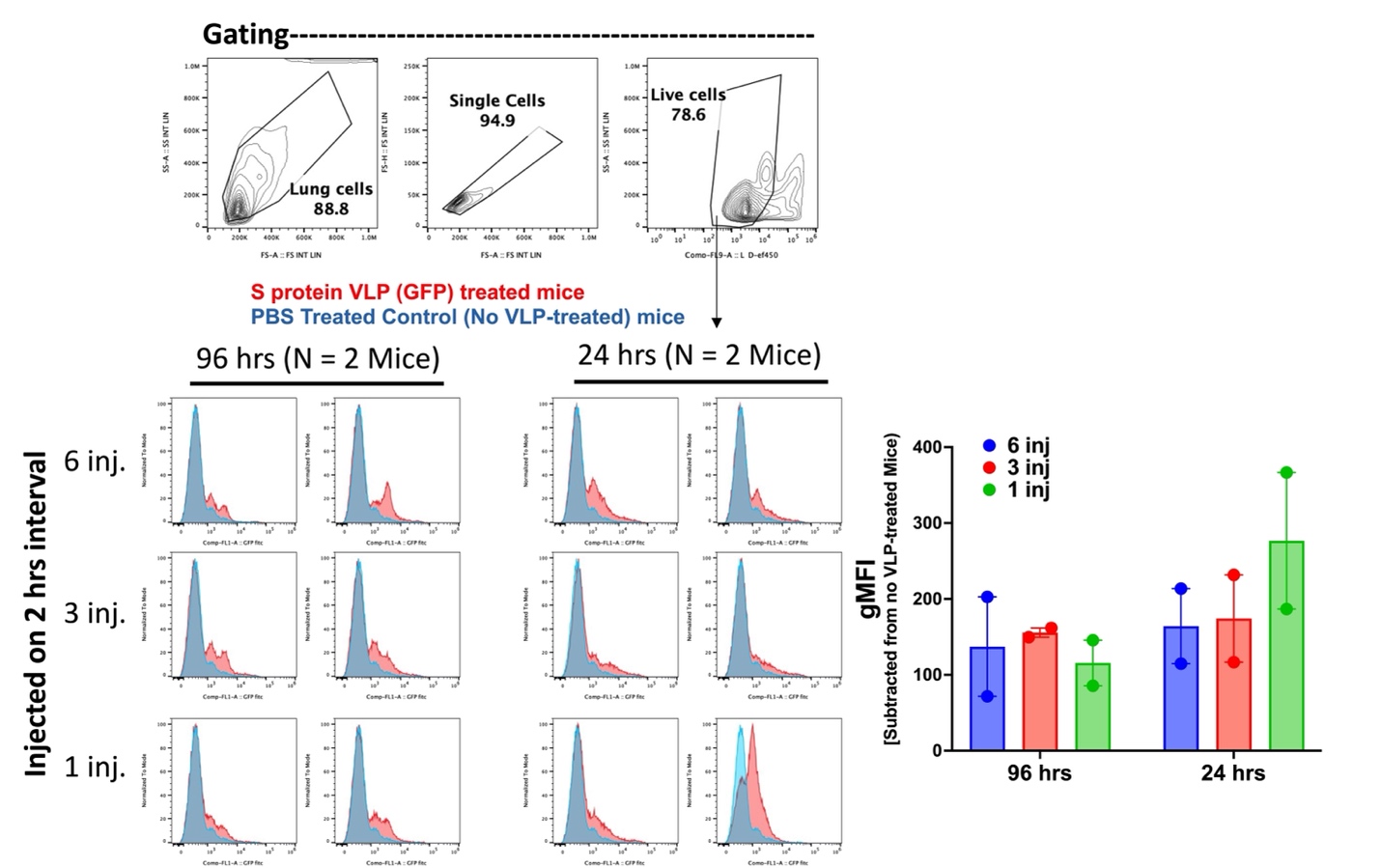


**Figure S2.** S protein mediated VLP uptake by lung cells of hACE2 Transgenic mice. 1x10^6^ VLP was administered to female B6.Cg-Tg(K18-ACE2)2Prlmn/J mice in 200 of DPBS buffer containing 1 mM MgCl_2_ via bronchoalveolar lavage on 2 hrs intervals as 1, 3, and 6 injections. Mice were sacrificed after 24 and 96 hrs, and lung cells were analyzed by flow cytometry to detect GFP signal from VLP in FITC channel. Individual dots represent data generated with cells from different mice.


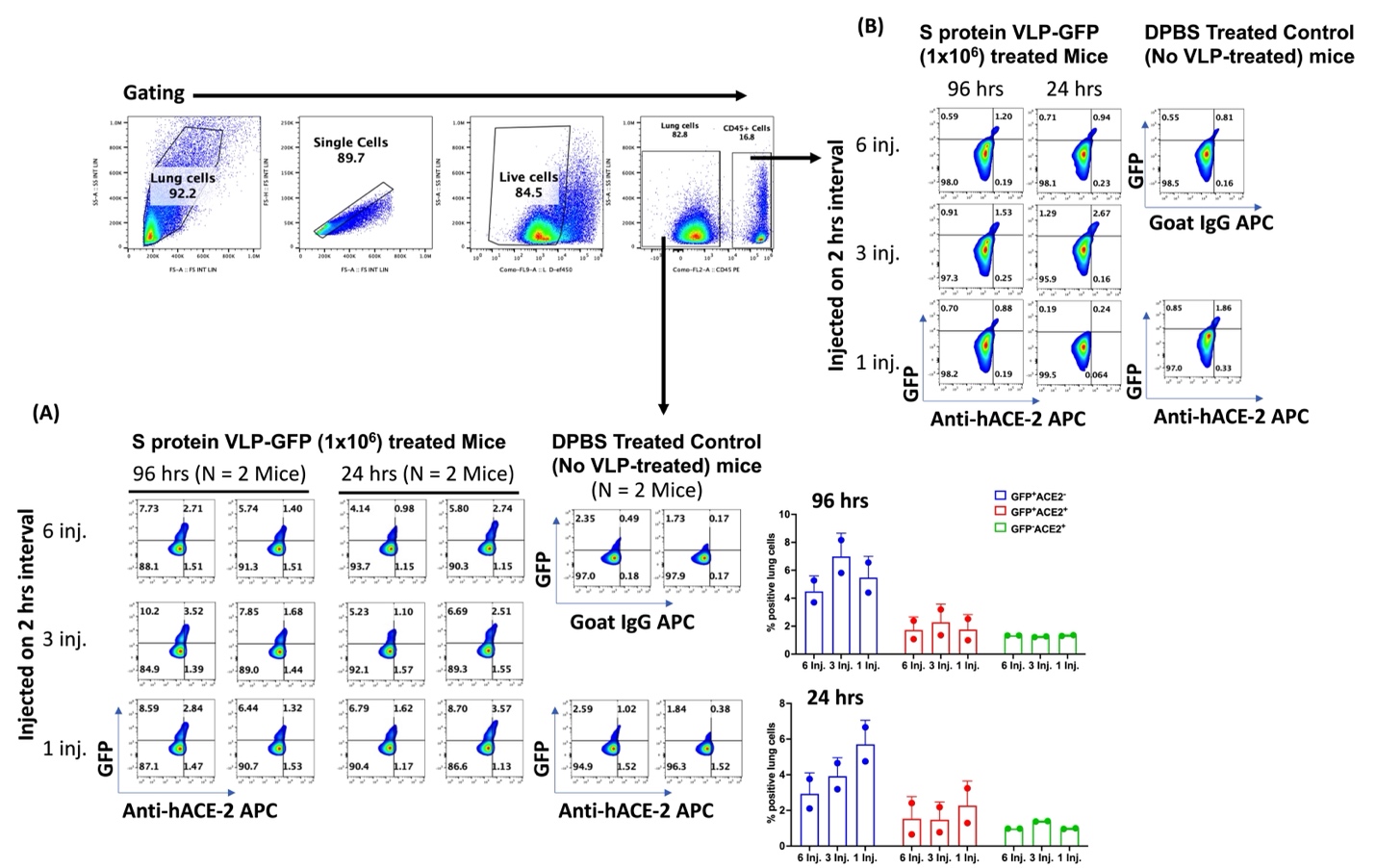


**Figure S3.** Expression of S protein VLP uptake by live lung cells (CD45- cells) in hACE2 Transgenic mice. Female B6.Cg-Tg(K18-ACE2)2Prlmn/J mice were administered with 1x10^6^ S protein VLPs in 200 of DPBS buffer containing 1 mM MgCl_2_ via bronchoalveolar lavage on 2 hrs interval as 1, 3 and 6 injections. Mice were sacrificed after 24 and 96 hrs; and cells were stained with Fixable Viability Dye, anti-mouse CD45, anti-human ACE2 and/or goat IgG. Cells were gated based on isotype staining (Goat IgG). Expression of hACE2 receptor and S protein VLP uptake by mice lung cells were assessed by flow cytometry analysis.


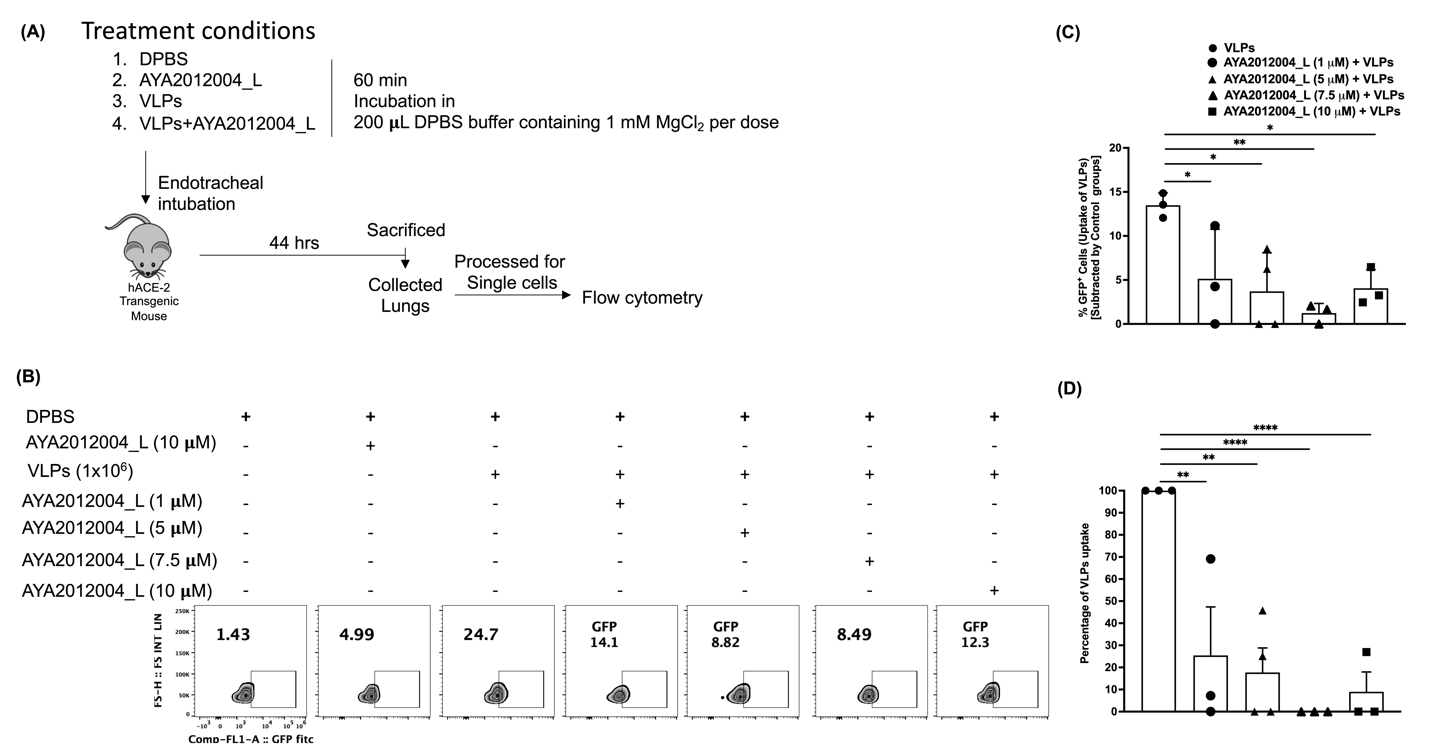


**Figure S4.** Aptamer induced dose-dependent neutralization of S protein VLP uptake by lung cells of hACE2 Transgenic mice. (A) Schematic experimental design of mice treatment with or without AYA2012004_L. (B) A representative FACS plot showing the neutralization effect of AYA2012004_L on the S protein VLP uptake by lung cells. Cells were gated based on DPBS treated group as control. (C) Summarized data for the frequency of GFP+ cells (uptake of S protein VLP) and (D) Summarized data for the percent uptake of S protein VLP by lung cells under every treatment condition. Uptake of S protein VLP by lung cells was calculated according to the formula: [(frequency of GFP+ cells of test group − frequency of GFP+ cells of control group)/(frequency of GFP+ cells of S protein VLP treated group − frequency of GFP+ cells of control group)] × 100. Individual dots represent data generated with cells from different mice (n=3). Data are expressed as mean ± SD. The p values were determined with one way ANOVA test (Dunnett’s multiple comparison test). *p<0.05, **p<0.01, ***p<0.001 and ****p<0.0001.

**
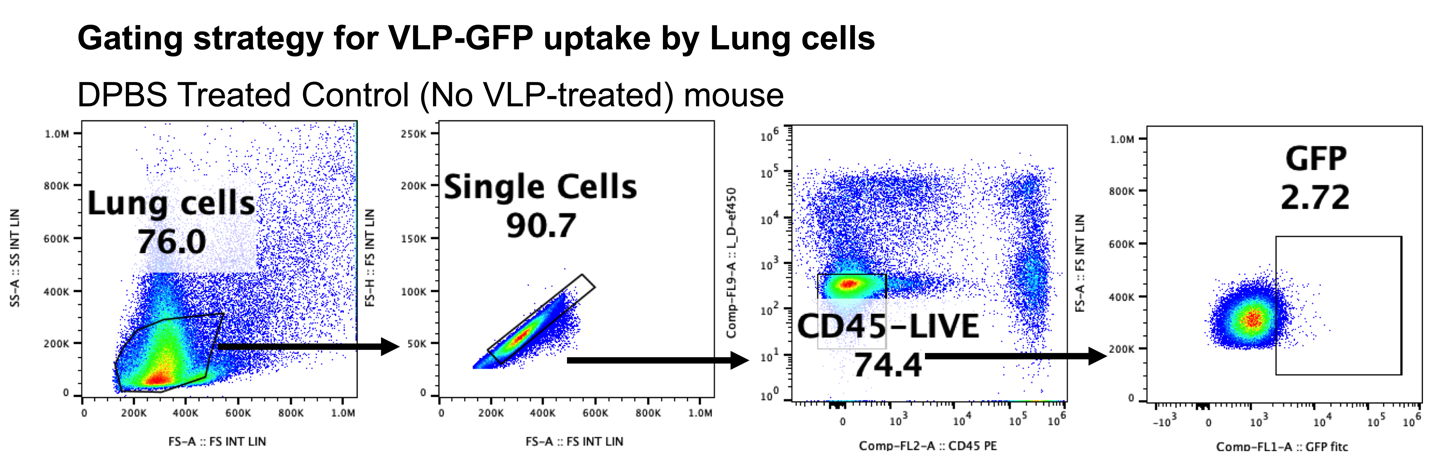
**

**Figure S5.** **Gating strategy for GFP-VLP signal.** Lungs were processed for single-cell isolation. Single cells from the lungs were gated for GFP signal (uptake of VLP-GFP) by the CD45^-^Live cells. Lung cells were stained with anti-mouse CD45 PE and fixable viability dye. Stained cells were acquired for three colors flow cytometry. Cells were gated first on FS-A and SS-A and then gated for single cells that were further gated for CD45^-^ Live cells (Lung cells). Live lung cells (CD45^-^ Live cells) were then gated from GFP. This gating strategy is applied in Figure 1 and Figure S5.


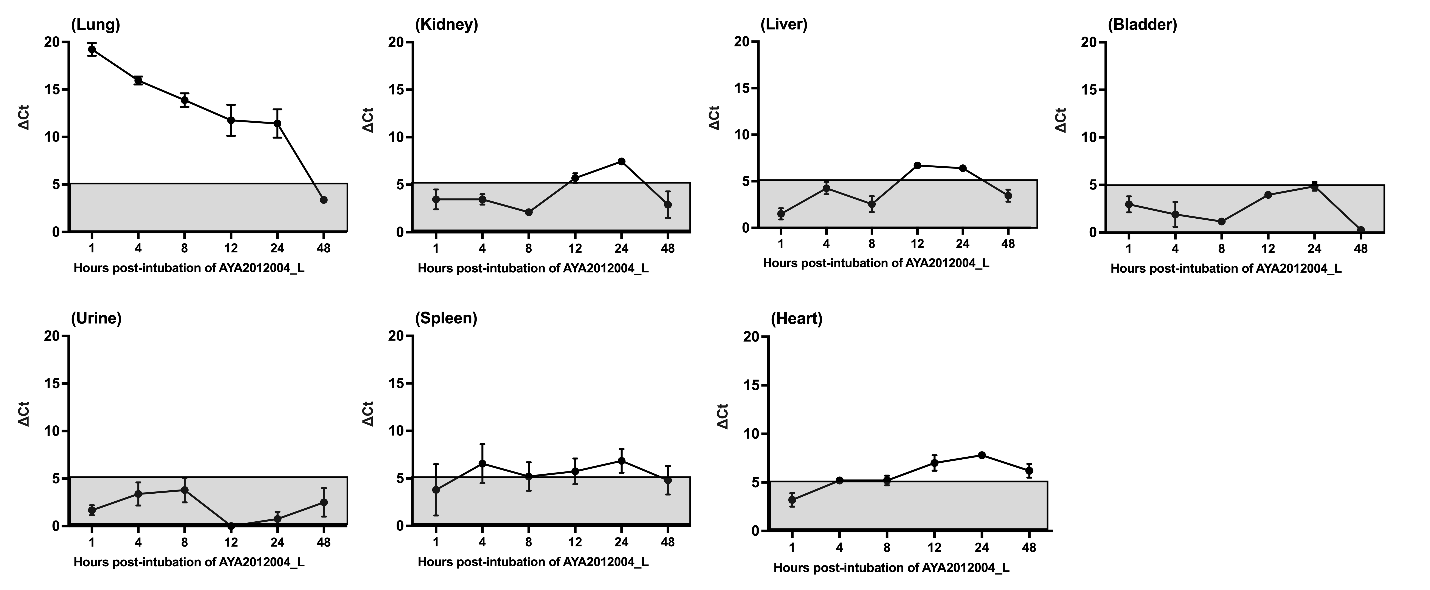


**Figure S6.** Detection of AYA2012004_L in mouse vital organs and urine by RT-PCR. C57BL/6J mice were administered with 200 μl of DPBS buffer containing 1 mM MgCl_2_ in the absence or presence of AYA2012004_L (10 μM) via bronchoalveolar lavage. Mice were then sacrificed at 1, 4, 8, 12, 24 and 48 hrs post-treatment. Lungs, livers, kidneys, hearts, spleens, bladder and urine were collected and processed for nucleic acid isolation. AYA2012004_L was detected in mice tissues by real time polymerase chain reaction (RT-PCR) assays. Grey color area is the cut off ∆Ct value in the organs of AYA2012004_L that demonstrates ≤0.02x10^9^ number of aptamer molecules per mg total DNA as calculated. The data are mean±SD of two independent experiments.


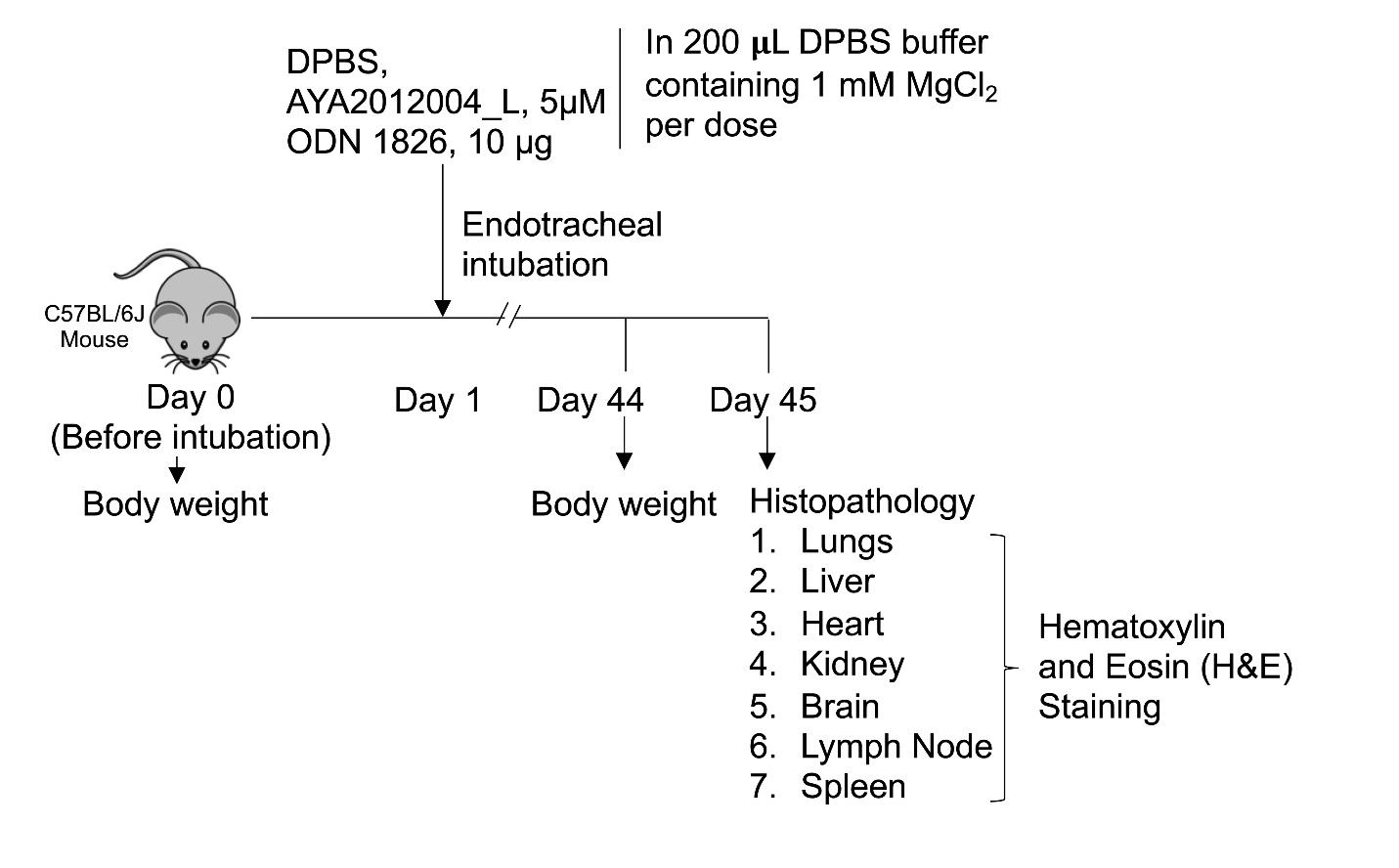


**Figure S7.** A schematic experimental design for histopathology analysis of mice tissues administered with 200 ul of DPBS buffer containing 1 mM MgCl_2_ in the absence or presence of AYA2012004_L (5 mM) or ODN 1826 (20 μg) at day 1 via bronchoalveolar lavage. Mice body weight were measured one day before treatment (on day 0) and on day 44 (24 hrs before study termination). On day 45, mice were euthanized, and tissues were harvested and processed for Haemotoxylin and Eosin (H&E).


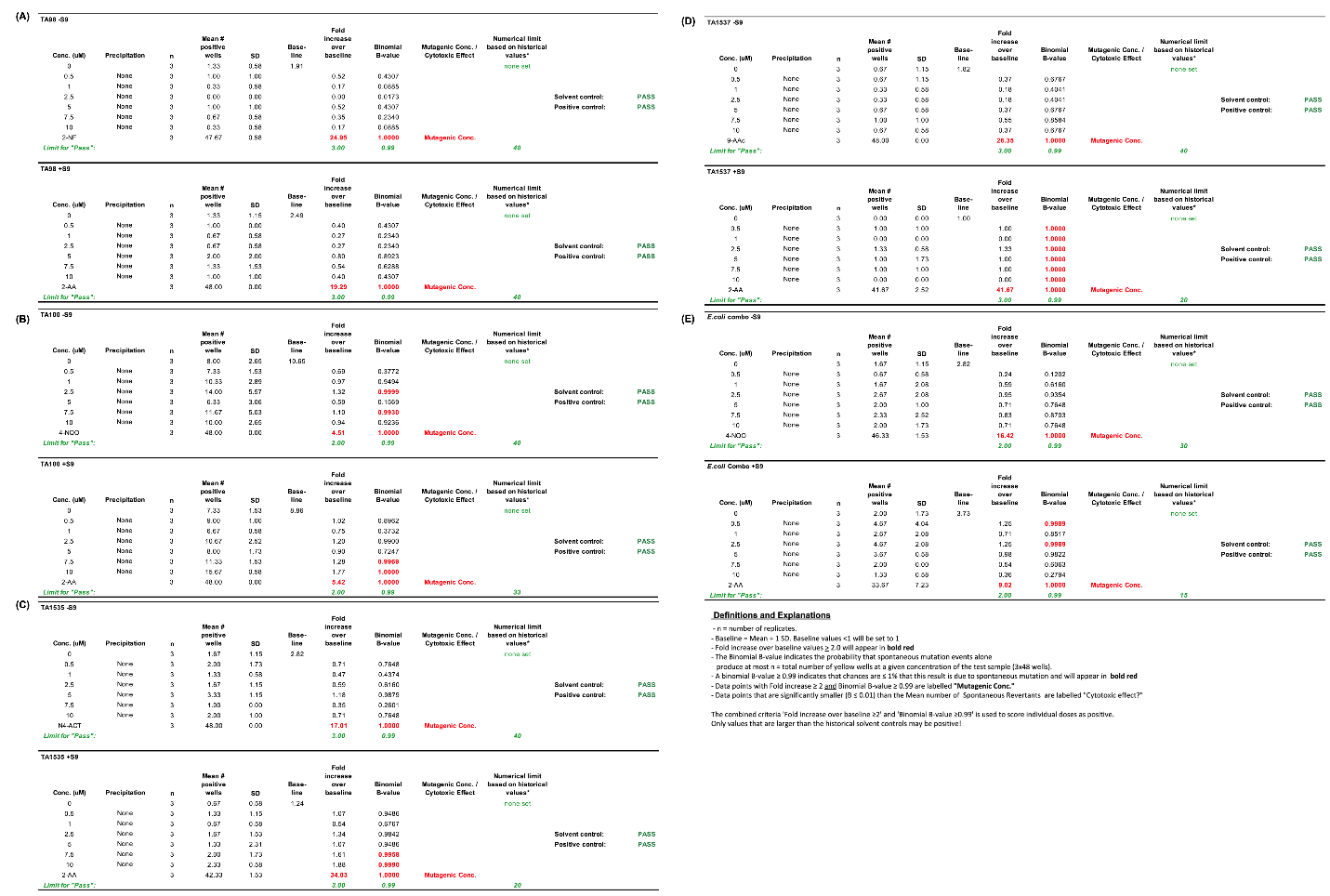


**Table S1.** Assessment of the mutagenic potential of AYA2012004_L.
